# AAV-mediated delivery of secreted acid **α**-glucosidase with enhanced uptake corrects neuromuscular pathology in Pompe mice

**DOI:** 10.1172/jci.insight.170199

**Published:** 2023-08-22

**Authors:** Naresh K. Meena, Davide Randazzo, Nina Raben, Rosa Puertollano

**Affiliations:** 1 Cell and Developmental Biology Center, National Heart, Lung, and Blood Institute, NIH, Bethesda, Maryland, USA.; 2 Light Imaging Section, Office of Science and Technology, National Institute of Arthritis and Musculoskeletal and Skin Diseases, NIH, Bethesda, Maryland, USA.

**Keywords:** Muscle Biology, Autophagy, Gene therapy, Skeletal muscle

## Abstract

Gene therapy is under advanced clinical development for several lysosomal storage disorders. Pompe disease, a debilitating neuromuscular illness affecting infants, children, and adults with different severity, is caused by a deficiency of lysosomal glycogen-degrading enzyme acid α-glucosidase (GAA). Here, we demonstrated that adeno-associated virus–mediated (AAV-mediated) systemic gene transfer reversed glycogen storage in all key therapeutic targets — skeletal and cardiac muscles, the diaphragm, and the central nervous system — in both young and severely affected old *Gaa*-knockout mice. Furthermore, the therapy reversed secondary cellular abnormalities in skeletal muscle, such as those in autophagy and mTORC1/AMPK signaling. We used an AAV9 vector encoding a chimeric human GAA protein with enhanced uptake and secretion to facilitate efficient spread of the expressed protein among multiple target tissues. These results lay the groundwork for a future clinical development strategy in Pompe disease.

## Graphical abstract

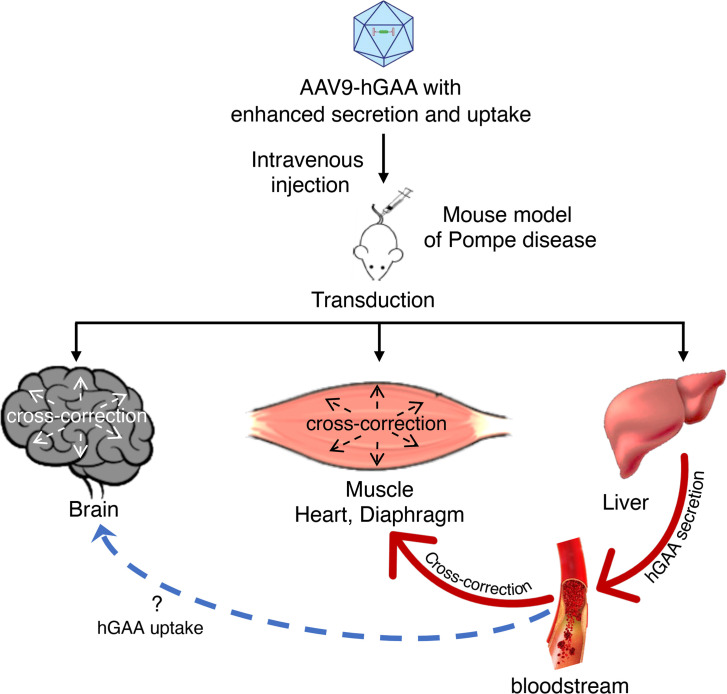

## Introduction

Glycogen storage disease type II, also known as Pompe disease (OMIM #232300), is an autosomal recessive neuromuscular disorder caused by deficiency of acid α-glucosidase (GAA), the enzyme hydrolyzing glycogen into glucose within lysosomes. Enzyme deficiency leads to accumulation of excessive glycogen and lysosomal dysfunction in tissues throughout the body, but clinical manifestations are dominated by cardiac and skeletal muscle involvement (1). The severe infantile-onset Pompe disease (IOPD), caused by a complete or near-complete lack of functioning enzyme, presents with hypertrophic cardiomyopathy, generalized muscle weakness, and death within the first year of life if left untreated (2). When the residual enzyme activity exceeds 1%–2% of normal, patients develop less severe juvenile- or adult-onset forms (referred to as late-onset Pompe disease; LOPD), in which cardiac muscle is usually spared, but slowly progressive limb and respiratory muscle weakness lead to ventilator and wheelchair dependency and shorter life expectancy (3).

More than 2 decades after its introduction, enzyme replacement therapy (ERT) remains the mainstay of treatment for Pompe disease. The initial clinical trials and multiple post-marketing studies with recombinant human GAA (rhGAA; Myozyme/Lumizyme; alglucosidase alfa; Genzyme Sanofi) have established the benefits and shortcomings of this approach. The therapy proved successful in rescuing life-threatening cardiac pathology and function, thus prolonging the lives of infantile patients (4–6). However, the therapy failed to reverse skeletal muscle abnormalities even at doses much higher (20–40 mg/kg) than recommended for ERT-based treatments of other lysosomal storage disorders. Most patients with LOPD experience a decline after initial improvements over the first few years on ERT (7, 8), and long-term survivors of infantile form experience muscle weakness along with emerging disease-related symptoms, including different degrees of cognitive decline due to cerebral white matter abnormalities (9–11). Two new drugs with enhanced content of mannose-6-phosphate (M6P) N-glycans that serve as a crucial signal for the cellular uptake and lysosomal trafficking of the recombinant enzyme via the cation-independent M6P receptor–mediated (CI-MPR–mediated) endocytosis (12, 13) — next-generation ERT with avalglucosidase alfa (already FDA approved; Nexviazyme, Genzyme) and cipaglucosidase alfa (Amicus Therapeutics) — have been recently tested against alglucosidase alfa in phase III clinical trials (14–16), and both showed modest improvement.

The waning therapeutic efficacy of ERT over time, combined with the inability of recombinant enzymes to cross the blood-brain barrier (BBB) and the need for frequent (most commonly every 2 weeks) lifelong intravenous infusions, make gene therapy a powerful option for Pompe disease treatment. Two main gene therapy strategies have been explored for Pompe disease: ex vivo lentiviral vector–based hematopoietic stem and progenitor cell gene therapy and in vivo adeno-associated virus (AAV) vector–mediated gene transfer (reviewed in refs. 17–19). Following rapid advances in basic science and extensive preclinical studies, the field has moved from proof of concept to clinical trials; so far, the trials have all been based on the AAV vectors for in vivo gene therapy. Several characteristics of these commonly used recombinant AAV vectors render them extremely appealing for gene therapy of inherited monogenic disorders affecting postmitotic tissues, such as their high efficiency of transduction of both dividing and nondividing cells, a wide tropism of their engineered serotypes, the ability of some serotypes to cross the BBB, and their excellent safety profile. Additionally, the recombinant AAV genome largely resides in the nucleus of transduced cells as nonreplicating episomes and rarely integrates into the host chromosome (20).

The first-in-human gene therapy trial (ClinicalTrials.gov NCT00976352) for Pompe disease, designed to address respiratory dysfunction, employed direct injection of recombinant adeno-associated virus acid alpha-glucosidase (rAAV1-CMV-GAA) into the diaphragm of a small group of children who required full-time invasive ventilation assistance despite ERT. The study demonstrated the safety of the AAV treatment, and although the clinical outcome was modest, this trial marks a major milestone in the field (21, 22). Ongoing clinical trials in patients with LOPD comprise intramuscular delivery of the GAA transgene (to test the feasibility of 2 administrations; NCT02240407; AAV9) and systemic delivery of AAV vectors carrying the transgene under the control of muscle- (NCT04174105; AAV8) or liver-specific (NCT03533673, AAV2/8; NCT04093349) promoters. The liver-targeted approach, which has gained a great deal of interest, harnesses the unique ability of the liver to induce tolerance (23) and produce and secrete hGAA into the circulation, thus providing a steady supply of the enzyme for “cross-correction” in skeletal and cardiac muscles. As with ERT, this approach relies entirely on the tissue uptake of the enzyme from the bloodstream. These promising gene therapy approaches are designed to correct muscle defects but do not address glycogen accumulation in the CNS, which has long been known as a feature in infantile patients based on autopsy reports (24, 25). However, the CNS involvement only recently became of particular concern in light of new clinical manifestations and results of neuropsychological tests in long-term survivors receiving ERT (10, 11, 26, 27).

Here, we investigated the efficacy of a rAAV9-based vector carrying an engineered *hGAA* under the control of a ubiquitous promoter (Amicus Therapeutics) in a large-scale preclinical study. The vector is optimized to express hGAA protein with enhanced secretion, and it incorporates a version of insulin-like growth factor 2 (IGF2) peptide to enable high-affinity binding to the bifunctional IGF2/CI-MPR (28) for enhanced tissue uptake and cross-correction. Our preclinical data show that this strategy efficiently rescued various aspects of muscle pathology and eliminated excessive glycogen accumulation in both muscle tissues and the brain.

## Results

In vivo efficacy of AAV9-mediated gene transfer was evaluated following a single intravenous administration of AAV9.CAG.BiP.vIGF2.hGAAco.rBG.KanR (systemic; SYS) vector (Amicus proprietary) into young (3–3.5 months of age) or old (8–9 months of age) *Gaa*-KO mice (referred to as KO), a model of Pompe disease (29). The rAAV9-based vector contains an engineered *hGAA* transgene that includes both an IGF2 tag (IGF2-hGAA) for enhanced uptake and a signal peptide from the binding immunoglobulin protein (BiP) for enhanced secretion; the transgene is driven by the CAG promoter (CMV early enhancer/chicken β-actin) to achieve systemic distribution among multiple tissues through transduction and cross-correction. To determine the optimal dose, we treated the animals at 0.5 × 10^13^ vector genomes/kg (vg/kg) (low dose), 5 × 10^13^ vg/kg (high dose), or 2.5 × 10^13^ vg/kg (intermediate dose). The tissues were harvested 1 month (short-term) or 6–8 months (long-term) after dosing. The experimental design is shown (Table 1). Considering the reported sex bias for liver-expressed AAV vectors in mice (30, 31), only KO males were used for the treatment to avoid variability in liver transduction.

### SYS vector dose optimization to achieve full correction of muscle pathology.

**Figure 1:**
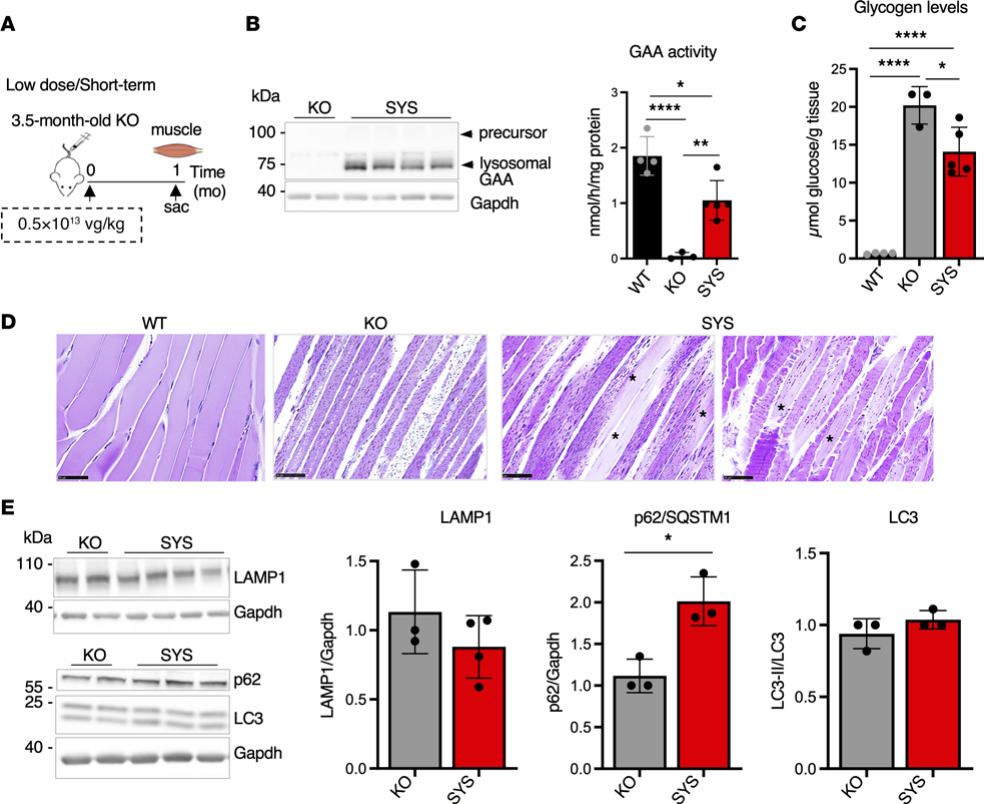
Systemic gene transfer fails to rescue muscle pathology after short-term treatment at a low vector dose. (**A**) Experimental design: 3.5-month-old KO mice received a single injection of systemic vector (SYS; *n* = 5) at a dose of 0.5 × 10^13^ vg/kg. Age-matched wild-type (WT) and untreated *Gaa*^–/–^ (KO) mice were used as controls. Muscle samples were collected 1 month (mo) after dosing. (**B**) Western blot analyses of whole muscle lysates with anti-human GAA antibody. Gapdh was used as a loading control. Graph shows GAA activity in muscle tissues from WT, KO, and SYS-treated KO mice. (**C**) Glycogen content in muscle tissues across the groups. (**D**) PAS-stained sections of gastrocnemius muscle; PAS-positive material (small dots) is seen in all fibers from KO mice; some fibers (or parts of a fiber) from SYS-treated KO mice appear normal (asterisks). Bars: 50 μm. (**E**) Western blot analyses of whole muscle lysates with the indicated antibodies. No significant decrease in the levels of lysosomal/autophagosomal markers is seen in treated compared to untreated KO. Statistical significance was determined by 1-way ANOVA and unpaired 2-tailed Student’s *t* test. Graphs represent mean ± SD. *P < 0.05; **P < 0.01; ****P < 0.0001.

SYS vector at a low dose (0.5 × 10^13^ vg/kg) was injected into 3.5-month-old KO, and skeletal muscle was analyzed 1 month after dosing (Figure 1A). The mature 76 kDa lysosomal form of hGAA protein was detected in muscle from treated mice (Figure 1B), but the enzyme activity did not reach physiological level and resulted in a modest, albeit significant, glycogen reduction compared with untreated KO (Figure 1C). Periodic acid–Schiff (PAS) staining for glycogen showed abundant PAS-positive structures in every fiber from untreated KO, whereas these structures were scarcer in fibers from SYS-treated animals, and some fibers appeared clear from excess glycogen (Figure 1D). The effect on autophagy — a major secondary abnormality in the diseased muscle (reviewed in refs. 32, 33) — was negligible. No significant changes in levels of LAMP1 (lysosomal marker), LC3 (autophagosomal marker; ref. 34), and autophagy-specific substrate SQSTM1/p62 (35, 36) were observed in treated compared to untreated KO (Figure 1E). Immunostaining of single fibers with LAMP1/LC3 showed enlarged lysosomes and LC3-positive areas of autophagic buildup in KO and most myofibers (88%; *n* = 126) from treated KO (Supplemental Figure 1; supplemental material available online with this article; https://doi.org/10.1172/jci.insight.170199DS1).

Similarly, the low dose did not fully rescue muscle pathology after long-term (6 months) treatment, though the effect was more pronounced compared with the short-term (Supplemental Figure 2, A–E). Thus, only partial improvement was achieved at a low dose, particularly long-term. Of note, a much higher efficacy of low-dose SYS gene transfer was observed in KO, in which autophagy was suppressed in skeletal muscle (DKO; ref. 37). After 1 month of therapy, muscle glycogen content in DKO was close to normal, underscoring the benefit of autophagy modulation to attain therapeutic significance at lower vector doses (Supplemental Figure 3).

**Figure 2:**
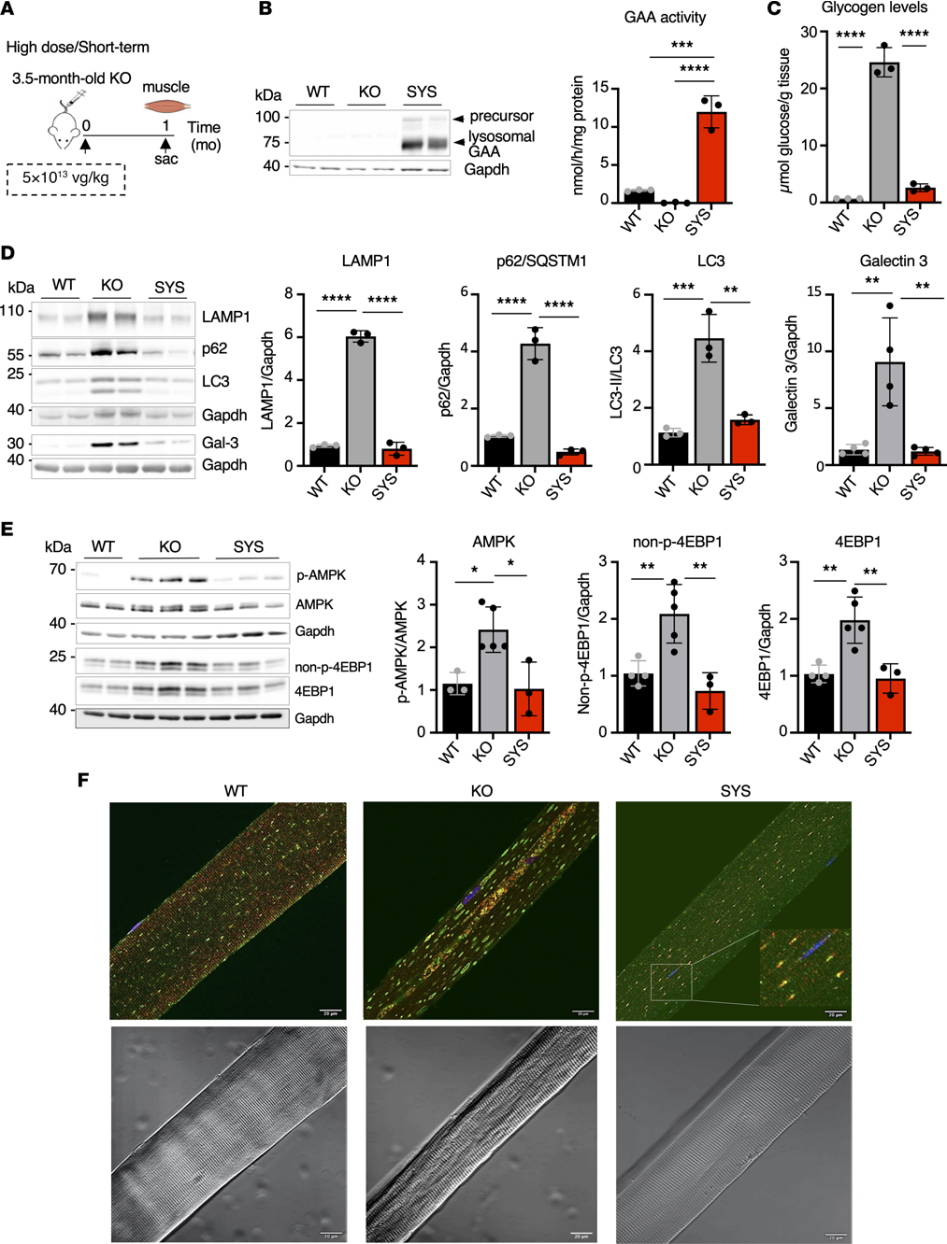
Systemic gene transfer rescues muscle pathology after short-term treatment at a high vector dose. (**A**) Experimental design: 3.5-month-old KO mice received a single injection of systemic vector (SYS; *n* = 3) at a dose of 5 × 10^13^ vg/kg. Age-matched wild-type (WT) and untreated *Gaa*^–/–^ (KO) mice were used as controls. Muscle samples were collected 1 month (mo) after dosing. (**B**) Western blot analyses of whole muscle lysates with anti-human GAA antibody. Gapdh was used as a loading control. Graph shows GAA activity in muscle tissues from WT, KO, and SYS-treated KO mice. (**C**) Glycogen content in muscle tissues across the groups. (**D** and **E**) Western blot analyses of whole muscle lysates with the indicated antibodies. Gapdh was used as a loading control. The treatment normalized the levels of lysosomal/autophagosomal markers (**D**) and AMPK activity (**E**). A significant decrease in both nonphosphorylated 4EBP1 (non-p4EBP1) and total 4EBP1 indicates an improvement in the mTORC1 signaling. (**F**) Top panel: Immunostaining of single fibers with markers for lysosomes (LAMP1; green), autophagosomes (LC3; red), and nuclei (Hoechst dye; blue); enlarged lysosomes and autophagic buildup (multicolored area in the core of the fiber) are seen in virtually all fibers from KO mice; lysosomes and autophagosomes appear as abundant dot-like LAMP1/LC3-positive structures (often located adjacent to each other; inset) in fibers from WT and SYS-treated KO mice. Bottom panel: Differential interference contrast images of the fibers shown on the top panel; ordered sarcomeric organization is fully restored after treatment. Statistical significance was determined by 1-way ANOVA. Graphs represent mean ± SD. *P < 0.05; **P < 0.01; ***P < 0.001; ****P < 0.0001. Bars: 20 μm. 4EBP1, eukaryotic translation initiation factor 4E-binding protein 1.

At a high dose (5 × 10^13^ vg/kg), remarkably, reversal of skeletal muscle pathology occurred in young (3.5-month-old) KO after 1 month on therapy (Figure 2, A–F). Western blot showed the lysosomal form of hGAA protein and enzyme activity far exceeded the WT values (Figure 2B), leading to glycogen reduction to near normal levels (Figure 2C). Treatment normalized levels of LAMP1, SQSTM1/p62, LC3, and LGALS3 (galectin, a marker of damaged lysosomes; refs. 38, 39) (Figure 2D); we previously reported a positive association of this marker with autophagic buildup in the diseased muscle (40). We have also examined the effect of a high dose on 2 major lysosome-linked signaling pathways, which reciprocally and antagonistically control autophagy — the energy sensor AMPK and growth-promoting mTORC1. Consistent with previous data (40–42), we found activation of AMPK and diminished mTORC1 activity in the diseased muscle in KO, as indicated by the increase in p-AMPK^Thr172^/AMPK ratio and level of non-p-4EBP1, an mTORC1-dependent translational inhibitor (43, 44) (Figure 2E). The treatment normalized p-AMPK^Thr172^/AMPK ratio, and the levels of total and non-p-4EBP1 fell significantly, reaching WT levels (Figure 2E). The vast majority of fibers from treated KO appeared normal, as shown by immunostaining of single muscle fibers with LAMP1 and LC3 (Figure 2F). Notably, at this high vector dose when the GAA activity reached supraphysiological levels, no deleterious effect was observed in skeletal muscle, and glycogen did not drop below physiological levels. Thus, SYS gene transfer at a high dosage completely reversed lysosomal glycogen accumulation, eliminated autophagic buildup, and improved AMPK/mTORC1 signaling after 1 month of therapy.

**Figure 3:**
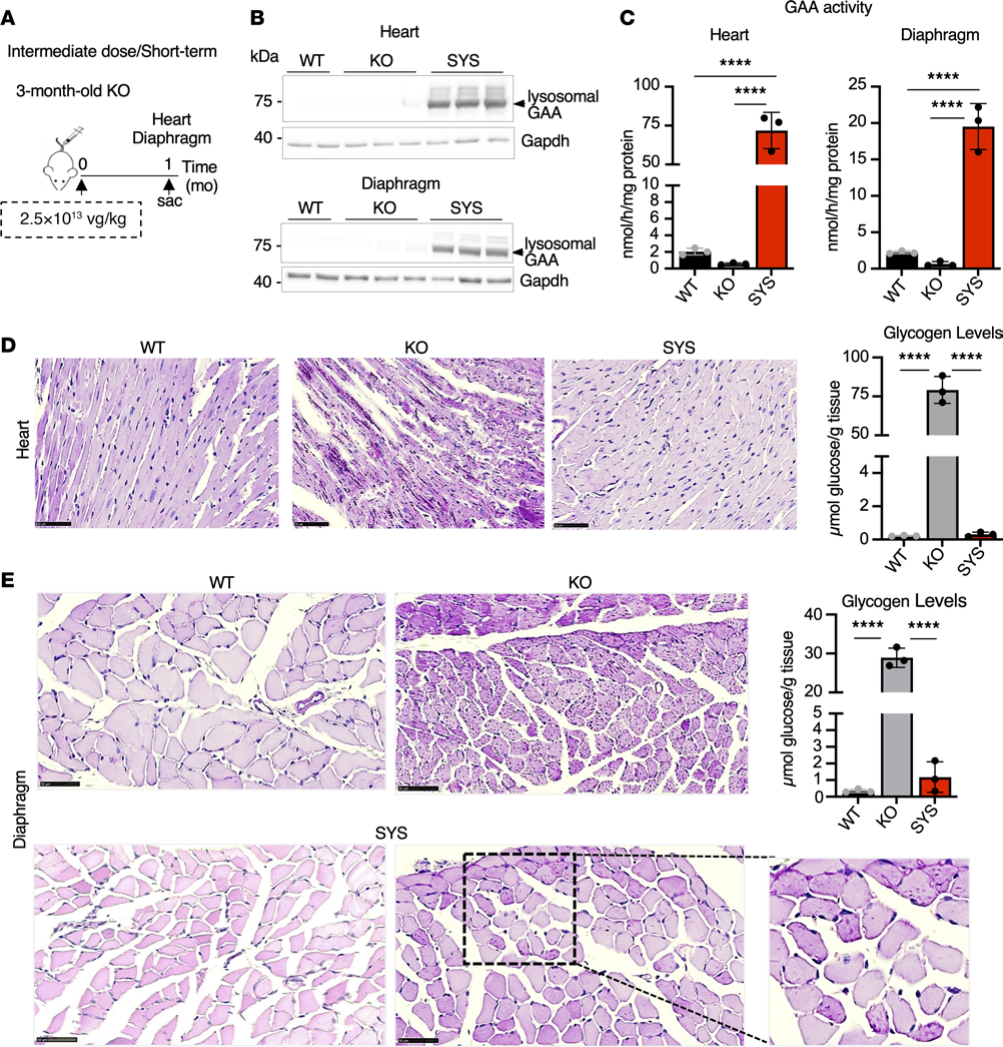
Systemic gene transfer rescues cardiac and diaphragmatic pathology after short-term treatment at an intermediate dose. (**A**) Experimental design: 3-month-old KO mice received a single injection of systemic vector (SYS; *n* = 3) at a dose of 2.5 × 10^13^ vg/kg. Age-matched wild-type (WT) and untreated *Gaa*^–/–^ (KO) mice were used as controls. Muscle samples were collected 1 month (mo) after dosing. (**B**) Western blot analyses of cardiac muscle and diaphragm lysates with anti-human GAA antibody. Gapdh was used as a loading control. (**C**) GAA activity in the heart and diaphragm far exceeds the WT levels. (**D** and **E**) PAS-stained sections of cardiac muscle and the diaphragm; PAS-positive material is abundant in the heart and diaphragm of a KO mouse; the pathology and glycogen content are fully normalized in the heart of SYS-treated KO; inset shows PAS-positive residual glycogen in some cells in the diaphragm despite the treatment. Bars: 50 μm. For inset in **E**, original magnification, ×2. Statistical significance was determined by 1-way ANOVA. Graphs represent mean ± SD. ****P < 0.0001.

Given that GAA activity in skeletal muscle at a high dose was far greater than the WT values (~8 times higher), we reasoned that a similar effect can be achieved at a lower dose. Indeed, SYS gene transfer at an intermediate dose of 2.5 × 10^13^ vg/kg resulted in complete glycogen clearance in muscle from 3-month-old KO after 1 month of dosing (WT: 0.73 ± 0.04 μmol glucose/g tissue; KO: 21.4 ± 1.98; SYS: 1.56 ± 0.39; *P* < 0.0001 SYS versus KO; not significant SYS versus WT). Furthermore, this dosage eliminated excess glycogen from the 2 other critical therapeutic targets — cardiac muscle and the diaphragm, in which GAA activity reached supraphysiological levels (Figure 3, A–E). Based on these short-term results, the intermediate dose was used in all subsequent experiments.

### IGF2-tagged hGAA secreted by the liver contributes to the efficacy of SYS gene transfer.

**Figure 4:**
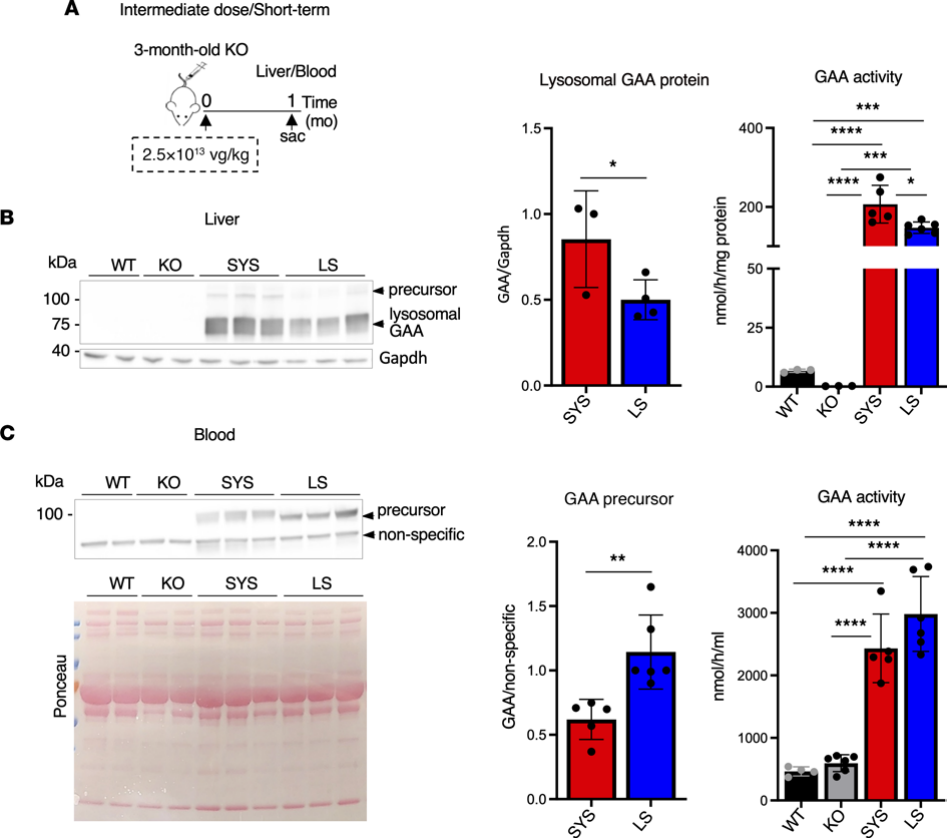
The transgene-encoded IGF2-hGAA precursor protein is secreted from the liver following systemic gene transfer. (**A**) Experimental design: 3-month-old KO mice received a single injection of systemic vector (SYS; *n* = 5) at a dose of 2.5 × 10^13^ vg/kg. AAV9-based liver-directed vector encoding untagged secretable hGAA served as a control (liver secreted, LS; *n* = 6). The samples were collected 1 month after dosing. (**B**) Western blot analysis of whole liver lysates with anti-human GAA antibody. Graphs show the amount and activity of the liver-expressed IGF2-tagged and untagged hGAA. (**C**) Western blot of plasma from SYS- and LS-treated KO mice. Ponceau stain was used to confirm equal protein loading. The amount of circulating precursor protein is quantified relative to a nonspecific band present in all samples. The amount and activity of the secreted hGAA are higher in LS- compared with SYS-treated KO mice. Statistical significance was determined by 1-way ANOVA and unpaired 2-tailed Student’s *t* test. Graphs represent mean ± SD. *P < 0.05; **P < 0.01; ***P < 0.001; ****P < 0.0001.

The efficacy in muscle tissues is expected to result not only from the transgene expression but also from the uptake of the circulating IGF2-hGAA protein secreted by the liver, since the universal CAG promoter drives the expression in multiple tissues, including the liver. Therefore, we analyzed enzyme levels and activity in the liver and blood following SYS vector administration at an intermediate dose (Figure 4). As a control, we used an AAV9-based vector — AAV9.TBG.Sp7.delta8hGAAco.rBG, LS (Amicus Therapeutics) — containing the liver-specific thyroxine binding globulin (TBG) promoter and *hGAA* transgene with Sp7 signal peptide (from the human α-1 antitrypsin enzyme) for enhanced secretion (45). Consistent with the previously reported difference in transduction efficiency between CAG and TBG promoters in the liver (46), Western blot showed a greater amount of lysosomal GAA protein and a higher enzyme activity in the liver from SYS- compared with LS-treated animals after 1 month of therapy (Figure 4, A and B). However, the amount of secreted precursor protein and GAA activity in blood appeared lower following SYS gene transfer, suggesting that Sp7 is more efficient than BiP signal peptide in driving liver secretion (Figure 4C). As expected, the size of the IGF2-tagged precursor protein in blood was larger than untagged protein (Figure 4C).

### Short-term SYS gene transfer at an intermediate dose rescues muscle pathology in young KO.

**Figure 5:**
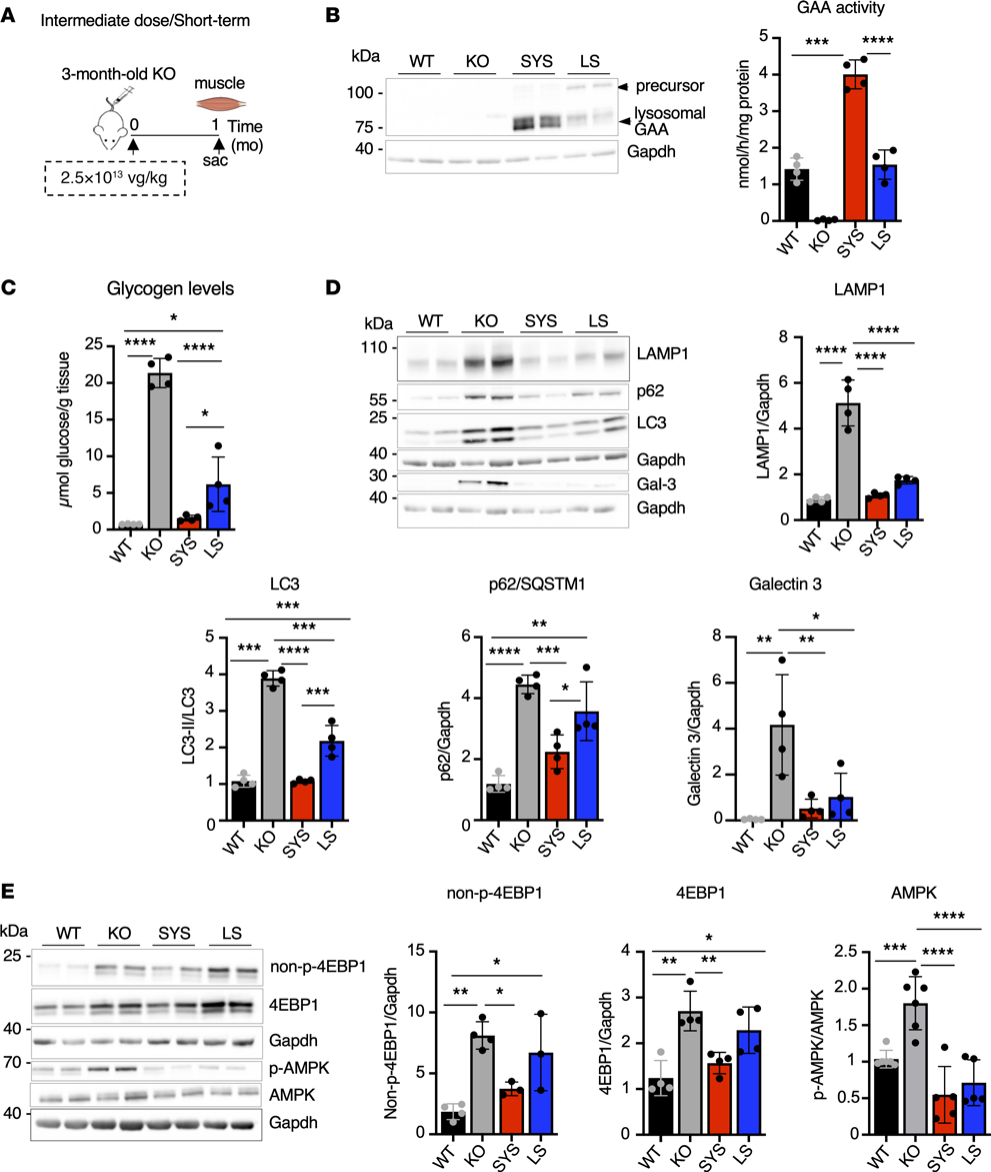
Muscle glycogen, autophagosomal-lysosomal markers, and mTORC1/AMPK signaling return to near normal after short-term systemic gene transfer at an intermediate vector dose. (**A**) Experimental design: 3-month-old KO mice received a single injection of systemic (SYS *n* = 4) or liver-secreted (LS *n* = 4) vector at a dose of 2.5 × 10^13^ vg/kg. Age-matched wild-type (WT) and untreated *Gaa*^–/–^ (KO) mice were used as controls. Muscle samples were collected 1 month (mo) after dosing. (**B**) Western blot analyses of whole muscle lysates with anti-human GAA antibody. The 110 kDa GAA precursor protein is clearly detectable after LS treatment. Gapdh was used as a loading control. Graph shows GAA activity in muscle tissues from WT, untreated KO, and KO treated with SYS or LS vector. (**C**) Glycogen content in muscle tissues across the groups. (**D** and **E**) Western blot analyses of whole muscle lysates with the indicated antibodies. Statistical significance was determined by 1-way ANOVA. Graphs represent mean ± SD. *P < 0.05; **P < 0.01; ***P < 0.001; ****P < 0.0001.

**Figure 6:**
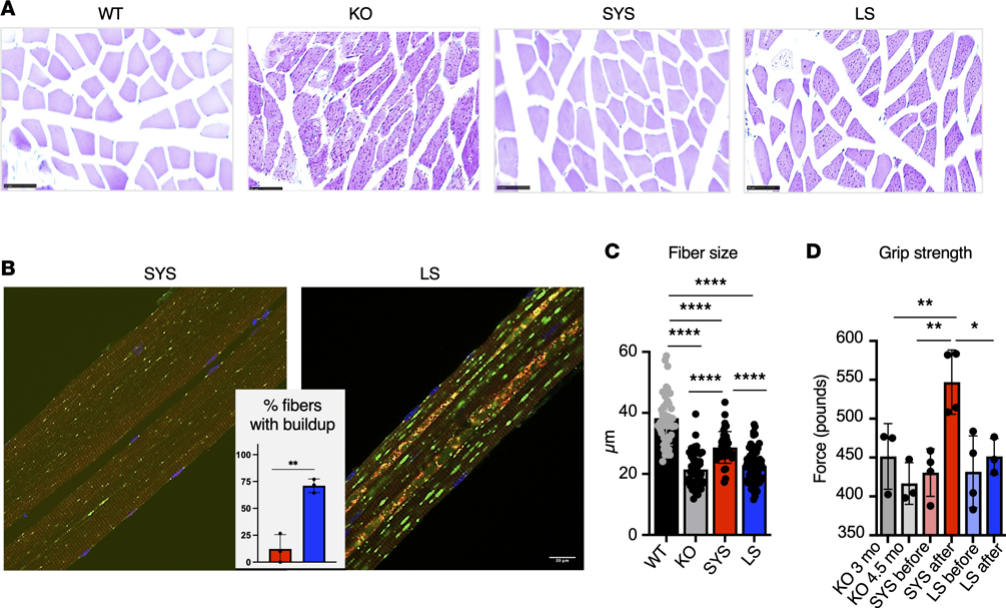
Systemic gene transfer rescues muscle pathology after short-term treatment at an intermediate vector dose. Experimental design is illustrated in Figure 5A. (**A**) PAS-stained sections of gastrocnemius muscle from WT, KO, and SYS- or LS-treated mice; PAS-positive material is seen in all fibers from KO and LS-treated mice (the staining is less intense in the latter); muscle fibers from SYS-treated mice appear normal. Bars: 50 μm. (**B**) Immunostaining of single fibers with markers for lysosomes (LAMP1; green), autophagosomes (LC3; red), and nuclei (Hoechst dye; blue); most fibers (88%) from SYS-treated KO mice (*n* = 3) are free from autophagic buildup; enlarged lysosomes and autophagic buildup (multicolored area in the core of the fibers) are seen in most fibers from LS-treated KO mice (*n* = 3). Bars: 20 μm. (**C**) Quantification of fiber size across the groups. (**D**) Muscle function was assessed using grip strength test before and after treatment (SYS, *n* = 4; LS, *n* = 4; KO, *n* = 3). Statistical significance was determined by 1-way ANOVA. Graphs represent mean ± SD. *P < 0.05; **P < 0.01; ***P < 0.001; ****P < 0.0001.

We then evaluated the effect of SYS and LS treatments at a dose of 2.5 × 10^13^ in muscle of 3-month-old KO (Figure 5 and Figure 6). This side-by-side comparison between SYS and LS vectors with similar viral load (Supplemental Figure 4) allowed us to assess the processing of the IGF2-tagged versus untagged hGAA. Although the levels of liver-secreted untagged hGAA protein were higher in blood, all outcome measures in muscle favored SYS treatment: greater amount of lysosomal GAA protein, supraphysiological GAA activity, and more efficient processing of the IGF2-tagged precursor (Figure 5B); better glycogen clearance (Figure 5C); and a larger reduction in the levels of autophagosomal-lysosomal markers (all reaching the WT levels, except for p62, which was significantly reduced) and proteins involved in mTORC1/AMPK signaling (Figure 5, D and E). Consistent with biochemical quantification of glycogen content, PAS-stained sections from SYS-treated mice were indistinguishable from the WT (Figure 6A), and the vast majority of myofibers (88%; *n* = 87 fibers as opposed to only ~30% with LS therapy; *n* = 153) were free from autophagic buildup (Figure 6B). Furthermore, SYS gene transfer resulted in a significant increase in muscle fiber size (at least 60 fibers were analyzed for each condition) and improvement of muscle strength after just 1 month on therapy (Figure 6, C and D). These results point to efficient processing and lysosomal targeting of IGF2-hGAA protein and establish therapeutic equivalence between intermediate and high vector dose in reversing all aspects of muscle pathology.

**Figure 7:**
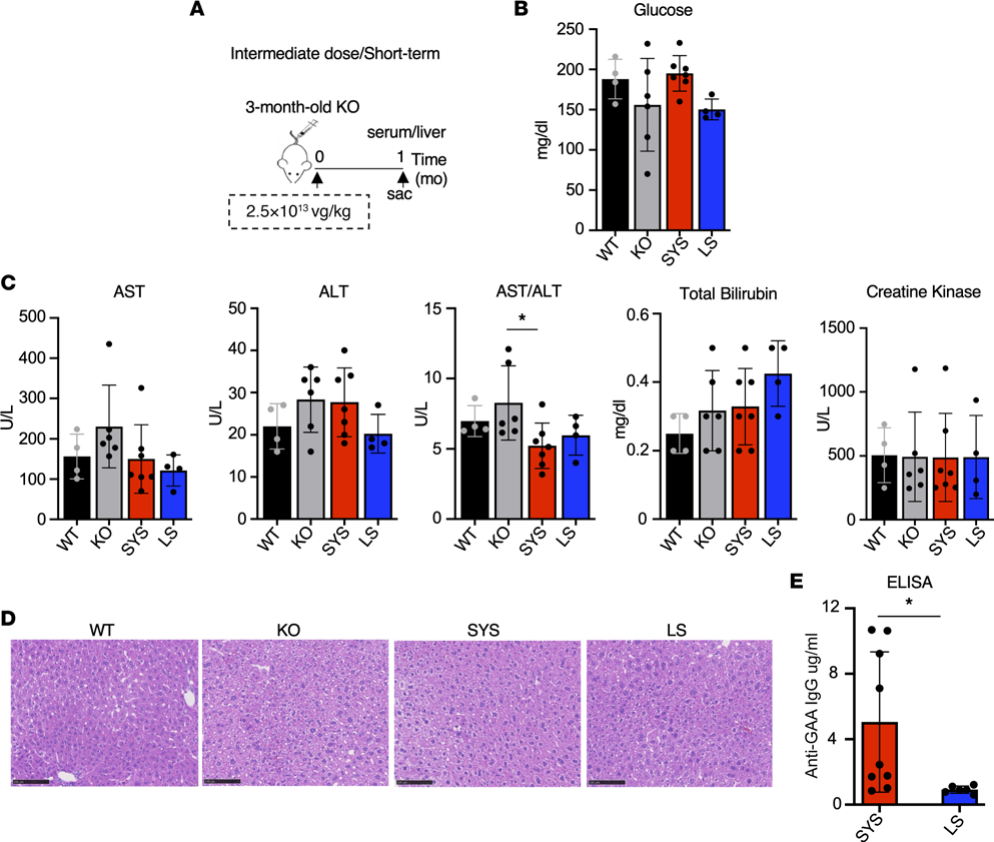
Systemic gene transfer does not affect blood glucose and liver function but induces low antibody levels against the transgene. (**A**) Experimental design: 3-month-old KO mice received a single injection of SYS or LS vector at a dose of 2.5 × 10^13^ vg/kg. Age-matched wild-type (WT) and untreated *Gaa*^–/–^ (KO) mice were used as controls. The samples were collected 1 month after dosing. (**B** and **C**) The levels of blood glucose and conventional hepatic serum biomarkers across the groups (SYS, *n* = 7; LS, *n* = 4). (**D**) Hematoxylin/eosin staining of liver sections. Bar = 100 μm. (**E**) Serum anti-human GAA IgG in SYS- (*n* = 9) and LS-treated (*n* = 6) KO mice. Statistical significance was determined by 1-way ANOVA and unpaired 2-tailed Student’s *t* test. Graphs represent mean ± SD. *P < 0.05.

To address a potential concern related to a pharmacologic effect of the IGF2 peptide on lowering blood glucose (47), we measured glucose levels in WT, KO, and SYS- and LS-treated KO mice in the same experimental setting (Figure 7A). The levels of blood glucose were no different among the groups, and all values remained within the normal range (Figure 7B and Supplemental Table 1). Yet another concern is related to hepatotoxicity and humoral immune response to the transgene-derived protein caused by high levels of the transgene expression in the liver. Serum liver enzymes (biomarkers), such as aspartate aminotransferase (AST), alanine aminotransferase (ALT), and total bilirubin, were all within the normal range in untreated and treated KO (Figure 7C and Supplemental Table 1). A moderate, statistically insignificant increase in AST/ALT ratio (a sign of hepatocellular injury) in untreated KO was reduced in treated animals. The levels of creatine kinase also remained normal across the groups (Figure 7C and Supplemental Table 1), and H&E staining of liver specimens did not show any signs of liver necrosis or inflammation (Figure 7D). Serum anti-hGAA IgG ranged from 0.85 μg/mL to 10.63 μg/mL in the SYS-treated group (*n* = 9), whereas the values were more consistent among the samples in the LS-treated group (0.6 up to 1.12 μg/mL; *n* = 6); overall, immunogenicity appeared higher in SYS-treated KO (*P* < 0.0367; Figure 7E and Supplemental Table 2).

### Complete rescue of muscle pathology in young KO is maintained long-term following SYS gene transfer.

**Figure 8:**
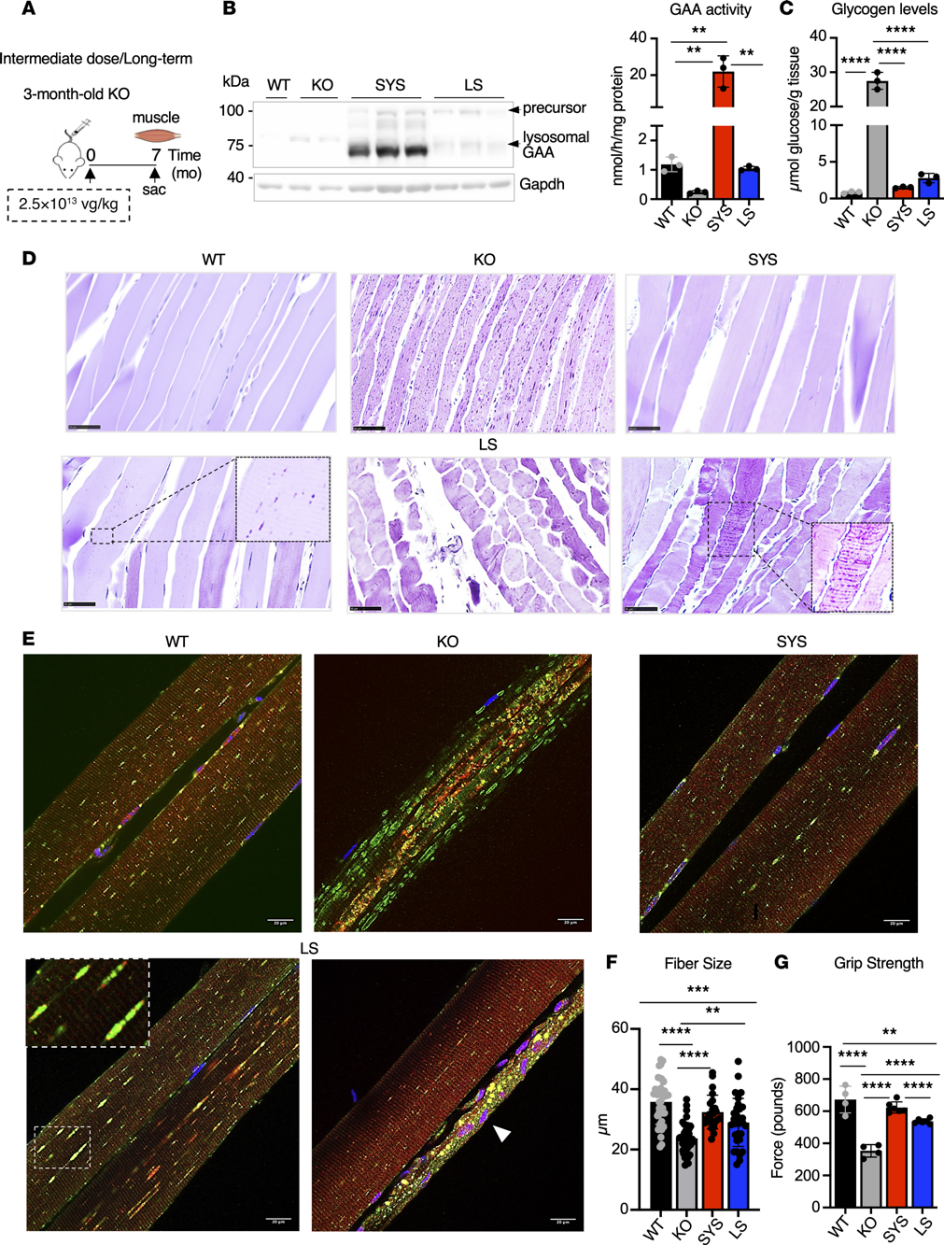
The efficacy of systemic gene transfer is sustained over long-term treatment. (**A**) Experimental design: 3-month-old KO mice received a single injection of SYS (*n* = 3) or LS (*n* = 3) vector at a dose of 2.5 × 10^13^ vg/kg. Age-matched (10- to 10.5-month-old) wild-type (WT) and untreated *Gaa*^–/–^ (KO) mice were used as controls. Muscle samples were collected 7 months after dosing. (**B**) Western blot analyses of whole muscle lysates with anti-human GAA antibody. Gapdh was used as a loading control. Graph shows GAA activity in muscle tissues from WT, untreated KO, and KO treated with SYS or LS vector. (**C**) Glycogen content in muscle tissues across the groups. (**D**) PAS-stained section of gastrocnemius muscle from SYS-treated mice appears normal (top right panel); individual fibers and clusters of fibers from LS-treated mice contain small PAS-positive material (bottom panels). Bars: 50 μm. (**E**) Immunostaining of single fibers with markers for lysosomes (LAMP1; green), autophagosomes (LC3; red), and nuclei (Hoechst dye; blue); muscle fibers from SYS-treated KO mice are free from autophagic buildup and appear normal; distended lysosomes and atrophic fibers (arrowhead) can be detected in fibers from LS-treated KO mice. Bars: 20 μm. Original magnification: **D** (PAS LS image on the left), ×2.5; **D** (PAS LS image on the right), ×1.5; **E** (LS), ×2.2. (**F**) Quantification of fiber size across the groups. (**G**) Muscle function was assessed using grip strength test after treatment. Statistical significance was determined by 1-way ANOVA. Graphs represent mean ± SD. **P < 0.01; ***P < 0.001; ****P < 0.0001.

Benefits of short-term SYS gene transfer were sustained 7 months after dosing, as indicated by the abundance of lysosomal hGAA protein, high levels of enzyme activity, and muscle glycogen clearance (Figure 8, A–C, and top of Figure 8D). Furthermore, long-term therapy fully rescued autophagic pathology (no LAMP1/LC3-stained fibers contained autophagic buildup; *n* = 73), normalized muscle fiber size, and restored muscle function (Figure 8, E–G). Of note, long-term exposure to the untagged LS hGAA also led to efficient muscle glycogen clearance (Figure 8, B and C), and the difference between the 2 groups became much more subtle; small PAS-positive structures (Figure 8D), mildly enlarged LAMP1-positive lysosomes, and a number of profoundly atrophic muscle fibers (3%–4%; *n* = 62) (Figure 8E) were detected in the LS-treated group, most likely contributing to incomplete restoration of muscle function (Figure 8G). The observation of a delayed effect of LS-untagged hGAA agrees with data showing that hepatic expression of hGAA and muscle exposure to hepatocyte-secreted enzyme are time dependent (48).

### SYS gene transfer completely rescues muscle pathology in severely affected old KO.

**Figure 9:**
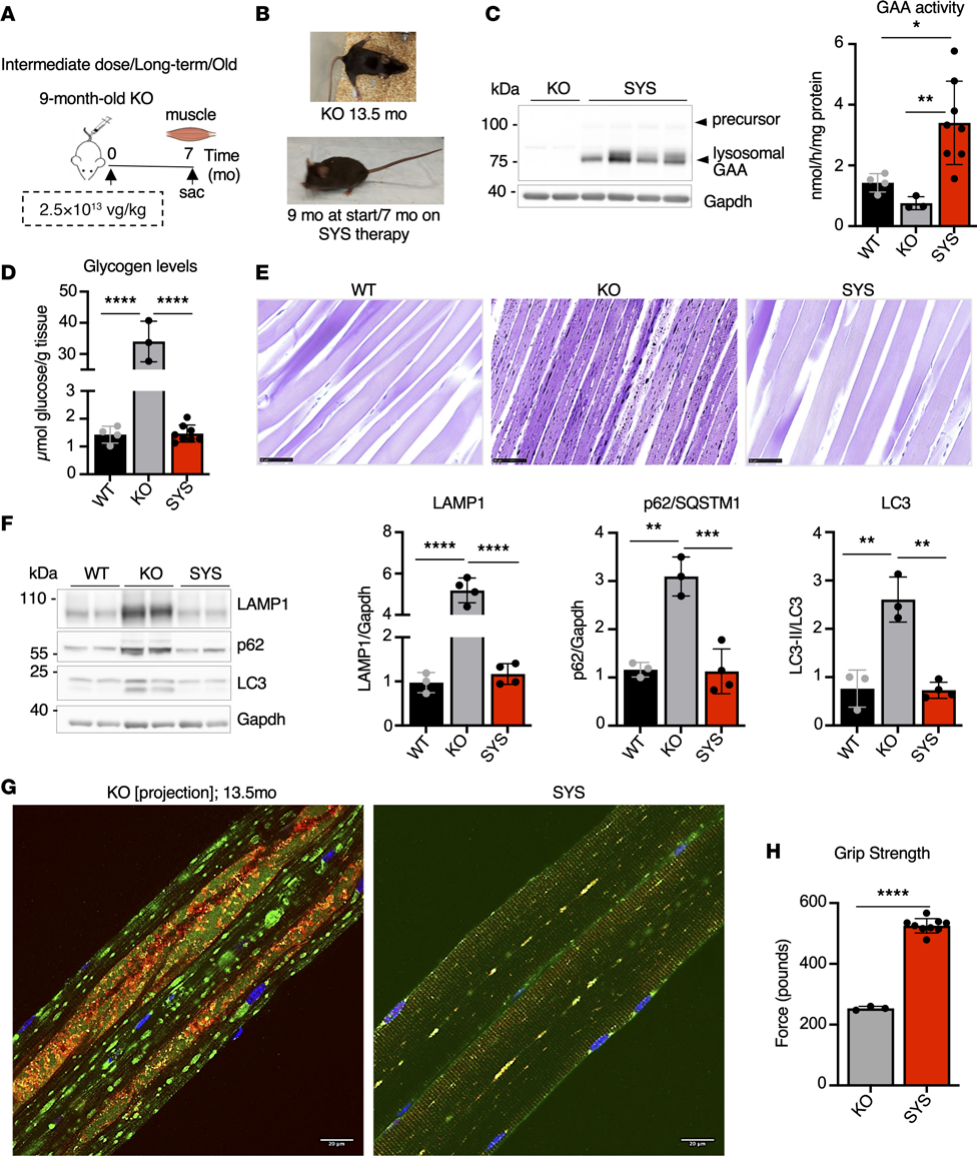
Systemic gene transfer reverses muscle pathology in old KO mice. (**A**) Experimental design: 9-month-old KO mice received a single injection of SYS (*n* = 8) vector at a dose of 2.5 × 10^13^ vg/kg. Age-matched (15.5-month-old) wild-type (WT) and 13.5-month-old untreated *Gaa*^–/–^ (KO) mice were used as controls. Muscle samples were collected 7 months after dosing. (**B**) SYS-treated KO mice appear healthy for their age group; a 13.5-month-old KO mouse shows profound muscle weakness and wasting; see Supplemental Videos 1 and 2. (**C**) Western blot analyses of whole muscle lysates with anti-human GAA antibody. Gapdh was used as a loading control. Graph shows GAA activity in muscle tissues from WT, untreated KO, and SYS-treated KO mice. (**D**) Glycogen content in muscle tissues across the groups. (**E**) PAS-stained sections of gastrocnemius muscle from WT, KO, and SYS-treated mice; muscle fibers from SYS-treated mice appear normal. Bars: 50 μm. (**F**) Western blot analyses of whole muscle lysates with the indicated antibodies. (**G**) Immunostaining of single fibers with markers for lysosomes (LAMP1; green), autophagosomes (LC3; red), and nuclei (Hoechst dye; blue); muscle fibers from SYS-treated KO mice are free from autophagic buildup. Bright autofluorescent particles consist of lipofuscin, a typical feature in patients and in a KO model of the disease (87); autophagic buildup in 13.5-month-old untreated KO occupies large portions of the fibers (the image shows projection view produced from 6 consecutive optical sections (*Z*-stack). Bars: 20 μm. (**H**) Muscle strength was assessed using grip strength test after treatment. Statistical significance was determined by 1-way ANOVA and unpaired 2-tailed Student’s *t* test. Data presented as mean ± SD; *P < 0.05; **P < 0.01; ***P < 0.001; ****P < 0.0001.

The remarkable efficacy of SYS gene transfer in restoring muscle function and pathology in young asymptomatic KO was replicated in old (9 months) KO (Figure 9, A–H). At the start of therapy, these mice show obvious clinical signs of muscle weakness and waddling gait (29, 49); the condition deteriorates progressively, and by the age of 13–14 months, the animals drag hind limbs and display profoundly wasted lower back muscle and kyphosis, necessitating euthanasia according to guidelines. Long-term SYS treatment (7 months) dramatically changed the disease course, and at the end of the follow-up (age 16 months) the KO appeared healthy, did not clench hind limbs to the body when suspended by the tail, and could stand on their hind limbs (Figure 9B; Supplemental Video 2). As in all the experiments described above, the 110 kDa precursor protein was hardly detectable, indicating efficient processing and lysosomal trafficking; the enzyme activity exceeded the WT levels, and muscle glycogen content returned to normal (Figure 9, C–E); autophagy markers were normalized, and autophagic area was detected in only occasional myofibers (~1.4%; 1 of 69) (Figure 9, F and G, and Supplemental Figure 5). All of these led to a markedly improved muscle function and phenotypic rescue (Figure 9H; Supplemental Video 2).

Thus, SYS gene transfer at 2.5 × 10^13^ vg/kg not only halted the progression of muscle damage but also provided a rapid and lasting reversal of muscle pathology in young and severely affected old KO. A comparative assessment of the effect of all treatments in skeletal muscle is shown in Supplemental Figure 6. Although GAA activity is not necessarily considered a good predictor of glycogen clearance in skeletal muscle (48, 50, 51), we saw a good correlation between these 2 variables, most likely because we routinely use a more homogeneous superficial part of gastrocnemius muscle, which predominantly consists of type IIB myofibers. The apparent correlation between the enzyme activity and glycogen clearance allowed us to establish a threshold of GAA activity that is required to rid muscle cells of excess glycogen. Muscle glycogen returns to normal, and the pathology is fully rescued only when the levels of GAA activity exceed the upper limit of the WT range. However, when GAA activity is below or even within the WT range, the reversal is not complete.

### SYS gene transfer rescues brain pathology.

**Figure 10:**
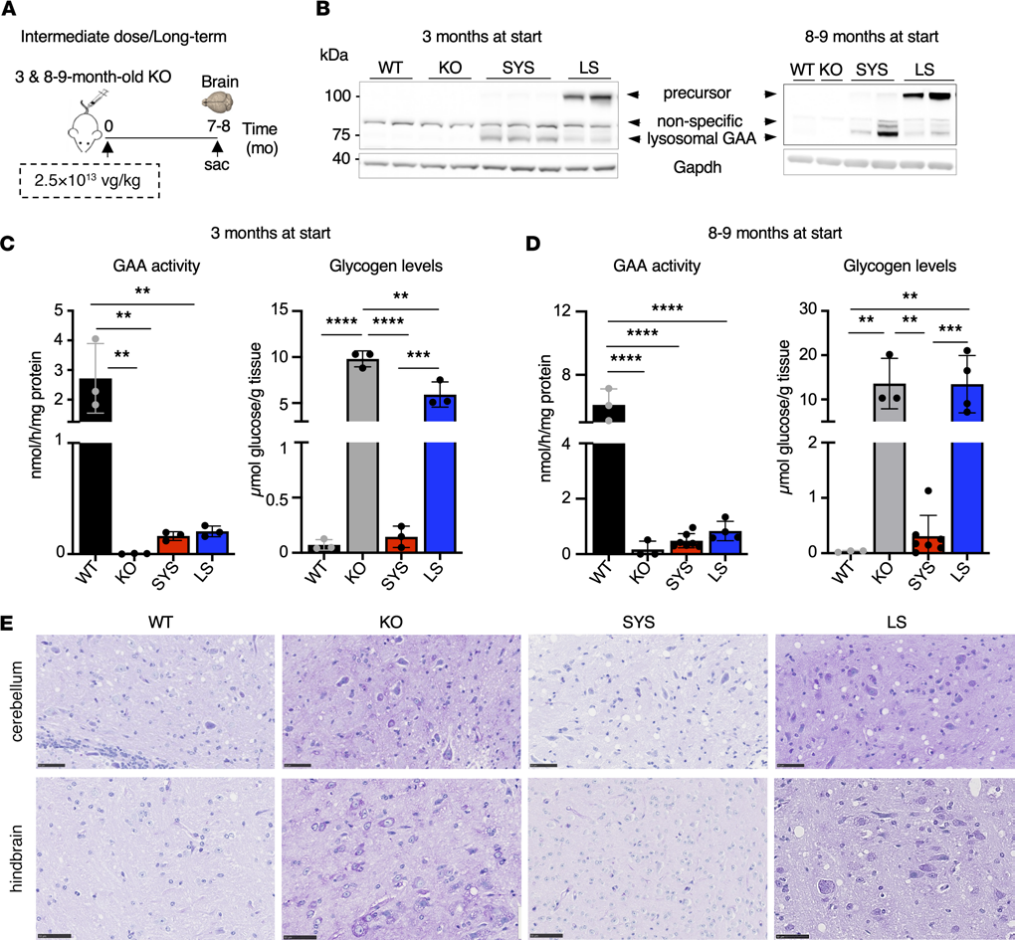
Systemic gene transfer restores glycogen accumulation to normal levels in the brain after long-term treatment at an intermediate vector dose. (**A**) Experimental design. Young (3-month-old) and old (8- to 9-month-old) KO mice received a single injection of SYS (*n* = 3 young; *n* = 7 old) or LS (*n* = 3 young; *n* = 4 old) vector at a dose of 2.5 × 10^13^ vg/kg. Age-matched wild-type (WT) and untreated *Gaa*^–/–^ (KO) mice were used as controls. The samples were collected 7–8 months after dosing. (**B**) Western blot analyses of whole brain lysates with anti-human GAA antibody. The 110 kDa GAA precursor protein is the predominant form in the brains of LS-treated KO mice. Gapdh was used as a loading control. (**C** and **D**) Graphs show GAA activity and glycogen content in brain tissues across the groups in young and old animals. (**E**) PAS-stained sections of brain tissues (cerebellum and hindbrain) across the groups in old animals; brain sections from SYS-treated mice appear normal; brain sections from LS-treated mice show glycogen storage in neurons and glial cells. Bars: 50 μm. Statistical significance was determined by 1-way ANOVA. Graphs represent mean ± SD. **P < 0.01; ***P < 0.001; ****P < 0.0001.

Glycogen accumulation in the CNS contributes to respiratory insufficiency and skeletal muscle dysfunction (52–55). Accumulating evidence indicates that glycogen storage in brain tissue is associated with signs of neurological deficit, most prominent in ERT-treated long-term survivors with IOPD (10, 11). Age-dependent glycogen storage in multiple regions of the brain in KO mice is also well documented (49, 56). Brain tissues from long-term SYS-treated young (3 months) and old (8–9 months) KO receiving intermediate dose were used for analysis. Again, LS-treated animals served as controls (Figure 10). Similar to what was observed in muscle, the amount of lysosomal GAA protein (which possesses optimal glycogen hydrolyzing activity) was greater in SYS- compared with LS-treated KO, but the enzyme activity, somewhat unexpectedly, was not different between the 2 groups (Figure 10, A–D). This apparent discrepancy is likely due to the abundance of the GAA precursor protein, which may contribute to the enzymatic activity in the brain of LS-treated KO (13, 57). In fact, the 110 kDa precursor was always detected in muscle on Western blots in samples from LS-treated mice; however, its abundance relative to the amount of lysosomal GAA was not as prominent as in the brain tissue. These data suggest that the IGF2-hGAA precursor expressed in the brain tissue (and, possibly, leaked across the BBB) is efficiently processed into fully active lysosomal form, whereas the processing of liver-secreted untagged hGAA precursor is very limited in brain tissue.

Consequently, only a modest glycogen reduction was observed in young but not old LS-treated mice (Figure 10, C and D). In contrast, SYS gene transfer resulted in efficient clearance of excess glycogen in both young and old animals, as shown by biochemical measurements and PAS staining (Figure 10, C–E). It was reported that CNS-targeted gene therapy in KO mice reverses or markedly improves neurological abnormalities when glycogen accumulation in the brain is maintained at normal levels (56, 58). Based on these results, it is reasonable to assume that SYS gene transfer had a similar effect. An interesting corollary of our studies is the demonstration that unlike skeletal muscle, the CNS pathology was reversed when the levels of enzyme activity were only approximately 10% of the WT values. Thus, SYS gene transfer resulted in robust reduction of glycogen accumulation in the heart, skeletal muscle, diaphragm, and CNS with values similar or close to those of WT animals.

## Discussion

Pompe disease has historically been viewed as an inherited metabolic myopathy, and the therapeutic efforts focused primarily on correcting skeletal and cardiac muscle abnormalities. Given the limited therapeutic efficacy of ERT in rescuing skeletal muscle pathology, multiple preclinical studies explored gene therapy using AAV vectors of different serotypes expressing GAA to deliver the transgene to skeletal muscle by local or systemic route (reviewed in refs. 17–19). The safety of the local (intradiaphragmatic) delivery was supported by the first clinical trial designed to improve respiratory insufficiency in ERT-treated patients with IOPD (21, 22). Systemic route has been explored in a recent preclinical study involving delivery of an AAV8 vector expressing GAA protein under the control of a muscle-specific promoter/enhancer (rAAV8-eMCK-hGAA; AT845). Complete glycogen clearance in the heart and quadriceps was achieved at a vector dose of at least 1 × 10^14^ vg/kg 3 months after administration into young 10- to 11-week-old KO mice (59). AT845 is currently being tested in a phase I/II clinical trial (NCT04174105; Astellas Gene Therapies). An alternative strategy is based on converting the liver into a biological factory for production and secretion of hGAA into the bloodstream; the newly synthesized secreted enzyme is recaptured by various organ tissues throughout the body in a process called cross-correction (60–62). This approach has gained increasing attention following the generation of AAV vectors containing a hepatocyte-specific promoter (hAAT) and an engineered transgene-encoded hGAA protein with enhanced secretion, thus providing sustained high levels of the enzyme in the circulation (AAV8-secGAA) (45, 63). Liver-directed gene therapy is now being tested in the ongoing clinical trial (NCT04093349; SPK-3006; Spark Therapeutics).

However, the paradigm shift in Pompe disease — which is now recognized as a neuromuscular disorder — underscores the limitation of these approaches. The CNS, along with cardiac and skeletal muscles, is emerging as a new therapeutic target in Pompe disease (reviewed in refs. 55, 64). Progressive white matter abnormalities and cognitive problems have recently come to light in ERT-treated infantile patients who survive much longer on ERT and reach adulthood (10, 11). Furthermore, preclinical studies demonstrated that targeted reversal of neuropathology via intracerebroventricular or intrathecal injection in KO mice resulted in improved motor function despite no reduction of muscle glycogen content (54, 58). The BBB, which is thought to be impermeable to lysosomal enzymes (except in the newborn period), was shown to be at least partially breached when high plasma activity of the enzymes was attained. Amelioration of CNS pathology was observed in a mouse model of mucopolysaccharidosis type IIIA following AAV-mediated liver-directed gene transfer (65). Liver-targeted gene transfer of AAV8-secGAA in young GAA-KO mouse models resulted in efficient glycogen clearance in cardiac and skeletal muscles and a modest clearance in the CNS (45, 48); partial reduction of glycogen content in the brain (~30%) was also achieved with the same AAV8-secGAA vector dose of 2 × 10^12^ vg/kg in old 9-month-old severely affected KO (66).

In this study, we have asked whether it is possible to develop a treatment that could reduce glycogen content in the CNS to normal level while preserving (or even improving) the ability to rescue muscle pathology. We have shown that AAV9-based SYS gene transfer can achieve this goal although at a higher dose of 2.5 × 10^13^. AAV9 serotype has been widely utilized in preclinical studies for Pompe disease gene therapy: AAV9 vector was used for systemic (67, 68), intramuscular (69), intralingual (70), and intrathecal (58) administration. The AAV9-based SYS vector, used in this study, combines the elements from those employed in Pompe clinical trials (AAV8-based NCT04174105 and NCT04093349) in that it provides transduction in muscle and high levels of LS GAA protein in the circulation. Importantly, the vector contains additional elements to afford some distinct advantages: 1) high tropism of AAV9 toward muscle and its ability to cross the BBB, thus enabling efficient transduction of multiple target tissues; 2) the transgenic expression of a chimeric IGF2-tagged hGAA with high affinity for the bifunctional IGF2/CI-MPR, thus allowing for efficient uptake from the circulation and cross-correction of the surrounding nontransduced cells within the tissue; and 3) the transgenic expression of IGF2-GAA linked to the secretion signal peptide (BiP; an HSP70 molecular chaperone) to direct the GAA precursor protein into the secretory pathway.

SYS gene transfer resulted in near-complete glycogen clearance in the brain of KO mice, indicating that this approach has a clear advantage in correcting the CNS pathology. Excess glycogen in the brain of KO mice is detectable at 1 month of age, followed by a striking increase by 3–4 months, and by the age of 12–15 months, the pathology is characterized as widespread (49, 56). A much-lower-than-normal level of GAA activity was shown to be sufficient to prevent glycogen buildup in the brain of KO mice treated at 1 month of age with intrathecal administration of AAV9-CAG-hGAA (58). Notably, we have shown that similarly low levels of GAA activity in the brain (<10% of the WT values) following SYS gene therapy were sufficient to reverse the fully established pathology in the brain of both young (3 months) and old (9 months) KO, suggesting efficient spread and cross-correction.

Similar rescue of the CNS pathology was achieved in 2-week-old KO following systemic administration of an AAV9 variant (called PHP.B) expressing hGAA under the control of CMV enhancer-chicken β-actin promoter at a dose of 5 × 10^12^ vg/kg (49, 56). Glycogen content was reduced to WT levels in the brain, heart, and quadriceps, but a significant amount of residual glycogen remained in the gastrocnemius muscle. An additional problem with this approach is that the exceptionally high CNS tropism of PHP.B capsid was shown to be limited to the model in which it was selected (a Cre-transgenic mouse on a C57BL/6J background) and was not replicated in other mouse strains or nonhuman primates (71). Normalization of glycogen levels in the heart, skeletal muscles, and the CNS, similar to what was found in this study, was recently demonstrated in the context of hematopoietic stem cell–mediated lentiviral gene therapy (HPSC-LVGT) for Pompe disease (50). However, unlike AAV gene therapy, lentiviral vectors (which integrate into the patient genome) have not yet been used in Pompe clinical trials.

The effect of SYS gene therapy in muscle tissues was equally impressive. In young KO, the treatment rescued muscle function and pathology — including excessive glycogen accumulation, autophagic defect, and mTORC1/AMPK signaling — just 1 month after the start of therapy. Importantly, these results were achieved using skeletal muscle (pale part of gastrocnemius), the tissue that is one of the most refractory to therapy (72). To the best of our knowledge, this outcome has never been attained in such a short time with any therapy for Pompe disease. The importance of rapid response cannot be overstated — shortening the time to halt the progression of the disease is critical to avert further age-dependent muscle deterioration. Moreover, even in old severely affected KO mice, long-term SYS therapy reduced muscle glycogen to the WT levels, fully reversed autophagic defect, normalized the size of the myofibers, and provided phenotypic rescue. At the age of 16 months, these SYS-treated KO appeared indistinguishable from the WT mice. A combination of muscle transduction, efficient secretion, cross-correction, and uptake of the enzyme from the circulation most likely account for this dramatic effect.

The BiP secretion signal peptide is a distinct element in the engineered IGF2-hGAA transgene. Although the efficacy of this signal peptide in driving secretion from the liver was somewhat lower than that of Sp7 (in a control vector for LS gene transfer), the liver-secreted IGF2-tagged hGAA precursor protein was readily detectable in the circulation, thus providing a steady supply of the enzyme for uptake in other tissues. (The difference between the efficacy of the 2 secretion peptides is not surprising because Sp7 is derived from the human α-1 antitrypsin enzyme, a protein that is physiologically highly secreted from the liver; ref. 45.) On the contrary, the addition of a short IGF2 fragment to the therapeutic lysosomal enzyme has been extensively tested as a strategy to provide a high-affinity ligand for CI-MPR through its interaction with a distinct IGF2 binding domain of CI-MPR (28, 73). M6P-capped lysosomal enzymes bind the sites in repeats 3, 5, and 9 within the 15 homologous repeats on the extracytoplasmic region of CI-MPR, whereas high-affinity IGF2 binding sites are in repeats 11 and 13 (74, 75). The benefits of the IGF2 peptide-based glycosylation-independent lysosomal targeting (GILT) strategy for ERT were first shown in a model of mucopolysaccharidosis type VII (28). Since then, this approach has been explored in several lysosomal storage disorders by generating IGF2-tagged recombinant enzymes for ERT or by incorporating this peptide tag into the viral cassettes for gene therapy.

IGF2-tagged lysosomal alpha-N-acetylglucosaminidase (NAGLU), the enzyme that is deficient in a severe neurodegenerative mucopolysaccharidosis type IIIB, was shown to normalize the enzyme activity in primary rat-derived neurons and astrocytes and in patients’ fibroblasts, whereas untagged rhNAGLU only normalized NAGLU activity in microglia (76). As for Pompe disease, targeted intralingual administration of an AAV9 encoding IGF2-tagged hGAA resulted in efficient glycogen clearance and reversal of pathology in lingual myofibers and hypoglossal (XII) motoneurons in a KO model (70); lingual weakness and hypoglossal neuropathology are common characteristics of Pompe disease (77–79). The therapeutic efficacy of the mentioned HPSC-LVGT in Pompe mice was much improved by the addition of IGF2 tag to the hGAA sequence (50). Furthermore, a chimeric GILT-tagged recombinant hGAA (Reveglucosidase alfa; BMN 701) for ERT was shown to reduce muscle glycogen deposits and improve respiratory function much more efficiently than untagged recombinant hGAA in KO mice (73, 80). Of note, the pharmacokinetics, safety, and exploratory efficacy of Reveglucosidase alfa were assessed in a phase I/II clinical trial in ERT-naive patients with LOPD. The patients showed improvement in respiratory function and mobility after 72 weeks of treatment (47). However, a small subset of patients developed transient hypoglycemia shortly after the drug administration, which was attributed to a pharmacologic effect of the IGF2 moiety. Importantly, in this study, we used a version of IGF2 peptide (Amicus proprietary) with amino acid substitutions to avoid off-target binding. Nonetheless, as a precaution, we did measure blood glucose levels and found no changes in SYS-treated KO.

Although the relatively high SYS vector dosage that is required for therapeutic efficacy is appropriate for clinical application (59, 68), a potential concern is the induction of antitransgene immune response. In fact, we did see an increase in anti-hGAA IgG in serum from KO mice treated with SYS and LS vectors at a dose of 2.5 × 10^13^ vg/kg. The antibody titers were greatly variable among individual animals within the SYS-treated group, resulting in a higher average value compared with the LS-treated group. These higher titers are comparable to those detected in the KO mice, which were treated with liver-directed AAV8 vector encoding the native GAA at a dose of 2 × 10^12^ vg/kg (45). However, the immune response was transient, and it disappeared 3 months after liver gene transfer (45), suggesting that the higher immunogenicity following SYS treatment may be transient as well. Furthermore, the increased levels of anti-hGAA IgG in some SYS-treated KO mice (up to ~10 μg/mL in 4 of 9 KO) were much lower than those reported in adult KO mice (up to ~80 μg/mL) following administration of AAV9 vector (at a dose of 2 × 10^12^ vg/kg) encoding native hGAA under the control of ubiquitous CAG promoter (81). These data suggest that the transgene-encoded IGF2-hGAA protein with enhanced secretion (secretable) is less immunogenic than native GAA, and that even at a higher vector dose the expression of the transgene in the liver following SYS gene transfer may dampen antitransgene immune response. However, further studies are needed to evaluate the long-term impact of the anti-hGAA antibody in SYS-treated animals. Another consideration is a risk of hepatotoxicity due to the high level of the transgene expression driven by a strong promoter. Although no elevations of conventional serum biomarkers that reflect alterations in hepatic function were detected following SYS gene transfer, additional monitoring is still needed to confirm the long-term safety of this approach.

Taken together, the data indicate that a single administration of SYS vector can rapidly and efficiently rescue the pathology in all key therapeutic targets in Pompe disease, including the CNS. In light of the remarkable therapeutic efficacy of the recently FDA-approved AAV9-based gene therapy (Zolgensma) for a severe motor neuron disorder, spinal muscular atrophy type 1 (82, 83), our findings may open the door for systemic gene therapy for Pompe disease.

## Methods

### Animal models, experimental design, treatment, and tissue processing.

Two animal models of Pompe disease were used: a *Gaa*^–/–^ (KO) strain carrying a targeted deletion of exon 6 (29) and muscle-specific autophagy-deficient *Gaa*^–/–^ (MLC-Cre Atg7^fl/fl^ *Gaa*^–/–^; DKO) strain, in which a critical autophagy gene, ATG7, was excised in skeletal muscle by Cre recombinase (37). AAV9.CAG.BiP.vIGF2.hGAAco.rBG.KanR vector (SYS) was used at 3 dosages: high (5 × 10^13^ vg/kg), intermediate (2.5 × 10^13^ vg/kg), and low (0.5 × 10^13^ vg/kg); AAV9.TBG.Sp7.delta8hGAAco.rBG vector (LS) was used at a dose of 2.5 × 10^13^ vg/kg. The vectors were provided under a Cooperative Research & Development Agreement (CRADA) executed between National Heart, Lung, and Blood Institute (NHLBI; NIH, Bethesda, Maryland, USA) and Amicus Therapeutics Inc. (Philadelphia, Pennsylvania, USA). We received consent from Amicus Therapeutics to make the virus (or plasmids) available to researchers; the Company agreed to provide us (NHLBI) and researchers with the plasmids to produce more virus. Graphical maps of the plasmids are shown in Supplemental Figures 7 and 8.

Male KO mice (*n* = 60) were randomly assigned to 3 groups (Table 1). Group 1 included young KO mice receiving a low dose of SYS vector for 1 (*n* = 5) and 6 months (*n* = 4). DKO mice (*n* = 6) were used for the evaluation of the effect of SYS vector at a low dose after 1 month of dosing. Group 2 included young (3.5-month-old) KO mice receiving a high dose of SYS vector (*n* = 3) for 1 month. Group 3 included both young (3-month-old) and old (8- to 9-month-old) KO mice receiving intermediate doses of SYS or LS vector. Young KO mice were treated for 1 month (*n* = 11 with SYS; *n* = 8 with LS) and 7 months (*n* = 3 with SYS; *n* = 3 with LS); old KO were treated for 7 months (*n* = 8 with SYS; *n* = 4 with LS). All animals were treated with a single injection of either vector in the tail vein (study day 0). Age-matched WT (*n* = 29) and untreated KO mice (*n* = 39) were used as controls. WT and untreated KO females were used in some experiments since no difference in the levels of GAA activity, glycogen content, and markers of autophagy is observed between the sexes in these strains. The resected tissues were either immediately snap-frozen in isopentane/liquid nitrogen and stored at −80°C until use for biochemical analyses or fixed for histological analysis and immunostaining of single muscle fibers.

### Measurement of GAA activity and glycogen levels.

GAA activity was measured by using 4-methylumbelliferyl-α-d-glucoside (MilliporeSigma) as the artificial fluorogenic GAA substrate as described (84). Briefly, muscle samples were homogenized in RIPA buffer (PBS containing 1% NP-40, 0.5% sodium deoxycholate, 0.1% SDS, and a protease/phosphatase inhibitor cocktail), sonicated, and centrifuged at 16,000*g* at 4°C for 15 minutes. The supernatants and plasma samples were diluted in distilled water; the diluted supernatants (10 μL) were incubated with the substrate (20 μL) in 0.2 M sodium acetate buffer (pH 4.3) for 1 hour at 37°C; the enzymatic reaction was stopped by adding 0.5 M carbonate buffer (pH 10.5). 4-Methylumbelliferone (MilliporeSigma) was used as a standard. Fluorescence was measured on a multilabel plate reader (Tecan, Spark 10M) at 350 nm excitation/460 nm emission. Protein concentration (BCA assay) was measured and used to normalize the data. Glycogen content was measured as the amount of glucose released after glycogen digestion with *Aspergillus niger* amyloglucosidase (MilliporeSigma) as described (85). Briefly, the diluted tissue lysates were denatured at 100°C for 3 minutes, then centrifuged at 9,000 rpm at room temperature for 3 minutes, and the supernatants were incubated with/or without 0.175 U/mL amyloglucosidase for 90 minutes at 37°C in 0.1 M potassium acetate buffer (pH 5.5) and boiled again to stop the reaction. The released glucose was measured using Glucose (Hexokinase) Liquid Reagents (Thermo Fisher Scientific) as recommended by the manufacturer; the absorbance at 340 nm was read on the Agilent Technologies Cary 60 UV-VIS Spectrophotometer.

### Western blot analysis.

For Western blotting, skeletal muscle (superficial part of gastrocnemius muscle), cardiac muscle, diaphragm, and brain tissues were homogenized in RIPA buffer and centrifuged for 15 minutes at 16,000*g* at 4°C. Protein concentrations of the supernatants were measured using the Bio-Rad Protein Assay, and equal amounts of protein were loaded on NuPAGE Bis-Tris protein gels (Thermo Fisher Scientific) in denaturing condition. Separated proteins were electrotransferred onto nitrocellulose membranes (Invitrogen). Membranes were then treated with blocking buffer (5% nonfat milk), incubated with primary antibodies overnight at 4°C, washed, incubated with the appropriate HRP-linked secondary antibodies (anti-rabbit IgG-HRP, 7074S; anti-mouse IgG-HRP, 7076S; anti-rat IgG-HRP, 7077S; Cell Signaling Technology), and washed again. HRP chemiluminescence was developed using Azure Biosystems Radiance Plus kit and scanned on the imager. The following primary antibodies (all diluted 1:1,000) were used: total and phosphorylated AMPK (2535; T172 5831; rabbit monoclonal) and total and nonphosphorylated 4EBP1 (4923; T46 9644, rabbit monoclonal) were from Cell Signaling Technology; LAMP1 (CD107a 553792; rat monoclonal; BD Transduction Laboratories), GAA (ab137068; rabbit monoclonal), SQSTM1/p62 (ab56416; mouse monoclonal), and GAPDH (ab9485; rabbit polyclonal) were from Abcam; and galectin-3 (sc-32790; mouse monoclonal) and LC3B (L7543; rabbit polyclonal) were from Santa Cruz Biotechnology and MilliporeSigma, respectively. Western blot of mouse plasma was performed on samples diluted 1:4 in RIPA buffer. Densitometry analysis was performed using Image Studio software.

### Immunostaining of single muscle fibers.

Gastrocnemius muscle was used for single muscle fiber analysis. Muscle fixation, isolation of single fibers, and immunostaining were performed as described (86) with some modifications. Briefly, muscle strips (pinned at their resting length on a Sylgard plate) were fixed with 2% paraformaldehyde (Electron Microscopy Science) in 0.1 M phosphate buffer for 30–40 minutes at room temperature. The strips were then removed from the plate, washed with PBS, incubated in cold methanol for 6 minutes at –20°C, washed again, and placed in 0.04% saponin in PBS on a Sylgard plate for manual isolation of single fibers under a dissecting microscope. The isolated fibers were stained with LAMP1 (1:200) and LC3 (1:500) antibodies using M.O.M. kit (Vector Laboratories). For each immunostaining and for confocal analysis, at least 25–30 fibers were isolated. The images were captured using a Zeiss LSM 780 confocal microscope with the Zeiss Efficient Navigation Black 2.3 software. Images were captured with a pixel format of 1,024 × 1,024 employing an oil immersion 40×/1.4 NA lens. Bright-field and fluorescence micrographs were acquired simultaneously using the following laser lines: 405 nm (for DAPI), 488 nm (for LAMP1), and 561 nm (for LC3), with pinhole size set at 1 airy unit and zoom set to 1.

### Anti-hGAA antibody detection.

Anti-hGAA IgG was detected by a standard ELISA. Briefly, 96-well plates (Thermo Fisher Scientific) were coated with 5 μg/mL rhGAA (Lumizyme) in coating buffer (10 mM phosphate buffer, pH 7.4; Invitrogen, CB07100). IgG standard curve was prepared using commercial IgG from mouse serum (MilliporeSigma, 18765). The plates were incubated overnight at 4°C, washed (wash buffer, Invitrogen, WB01), blocked at room temperature in blocking buffer (Thermo Fisher Scientific 37581, pH 7.4), and incubated with the serum samples (diluted 1:20 in blocking buffer) overnight at 4°C. HRP-conjugated goat anti-mouse IgG (whole; 1:5,000; MilliporeSigma, A4416) was used as a secondary antibody. The plates were then washed, incubated with the substrate, tetramethylbenzidine (TMB Substrate Solution, SB01, Invitrogen), for 1 minute at room temperature; the reaction was stopped by H_2_SO_4_ (stop solution, Invitrogen, CNB0011), and the plates were read at a wavelength of 450 nm using SpectraMax iD3 reader.

### Analysis of vector load in the liver.

Genomic DNA was isolated from the liver using QIAGEN DNeasy Blood & Tissue Kit. Viral load was measured by quantitative PCR (qPCR) using 75 ng DNA, nRBG (rabbit β-globin) primers, and a probe: forward TTCCCTCTGCCAAAAATTATGG; reverse CCTTTATTAGCCAGAAGTCAGATGCT; probe (6-FAM)-ACATCATGAAGCCCC-(MGBEcl-Q); the sequences were provided by Amicus Therapeutics. Viral load was normalized by the copy of GAPDH gene measured in each sample. The primers and a probe for GAPDH were as follows: forward GGCAAATTCAACGGCACAGT; reverse GGCCTCACCCCTTTGATTG; probe (6-FAM)-TCCAGGAGCGAGACCCCAC-(MGBEcl-Q). The qPCR was performed using TaqMan Fast Advanced 2× Master Mix (Applied Biosystems, 4444557). PCR conditions were 50°C for 2 minutes, 95°C for 10 minutes, and 40 cycles of 95°C for 15 seconds and 56°C for 20 seconds.

### Histology and measurements of blood glucose and liver enzymes.

For PAS staining, muscle tissues were fixed in 3% glutaraldehyde (EM grade, Electron Microscopy Sciences) in 0.2 M sodium cacodylate buffer for 4 hours at 4°C, washed in 0.1 M sodium cacodylate buffer, and stored at 4°C in the same buffer. For PAS staining of brain tissues, the samples were fixed in 10% neutral-buffered formalin (NBF) for 48 hours, postfixed in NBF containing 1% periodic acid for another 48 hours at 4°C, embedded in paraffin, sectioned, and stained with PAS by standard procedures. Liver tissues were fixed in 10% NBF, and serial sections were stained with hematoxylin and eosin according to the standard procedure. All stained slides were scanned and captured using a Hamamatsu NanoZoomer XR slide scanner equipped with a Plan Apochromatic 20×/0.75 NA lens (Olympus). The images were viewed, processed, and exported as.TIFF using the NDP.view2 software (Hamamatsu). The levels of glucose, bilirubin, creatine kinase, AST, and ALT in blood were measured according to the standard procedure (IDEXX BioAnalytics). The animals were starved overnight prior to the blood collection.

### Functional muscle strength test.

Muscle strength test was performed using a Grip Strength Meter (Columbus Instruments) according to the manufacturer’s recommendations. The test measures maximal combined forelimb/hind limb grip strength as an indicator of neuromuscular function. Briefly, the animal is placed on a wire grid and allowed to grasp it; the animal’s base of the tail is then gently pulled backward horizontally until the grasp is released. The strength is measured by the grasping applied by the mouse on a grid that is connected to a monitoring device. The force applied to the grid is recorded as the maximal peak force displayed by the animal. Each mouse was tested 3 times with 10- to 15-minute intervals per session; 3 sessions were conducted over 3 consecutive days.

### Statistics.

Statistical significance was calculated by using GraphPad Prism software. One-way ANOVA and unpaired 2-tailed Student’s *t* test were performed. Data are presented as mean ± SD; *P* < 0.05 was considered statistically significant.

### Study approval.

Animal care and experiments were conducted in accordance with the NIH *Guide for the Care and Use of Laboratory Animals* (National Academies Press, 2011). The study was approved by the NHLBI Animal Care and Use Committee.

### Data availability.

Values for all data points shown in graphs can be found in the Supporting Data Values XLS file.

## Author contributions

NKM performed experiments and analyzed and interpreted the data; DR performed imaging experiments and analyzed and interpreted the data; NR designed the study, performed experiments, analyzed and interpreted the data, and wrote the paper; RP supervised the project, analyzed and interpreted the data, and contributed to writing the manuscript.

## Supplementary Material

Supplemental data

Supplemental video 1

Supplemental video 2

Supporting data values

## Figures and Tables

**Table 1:**
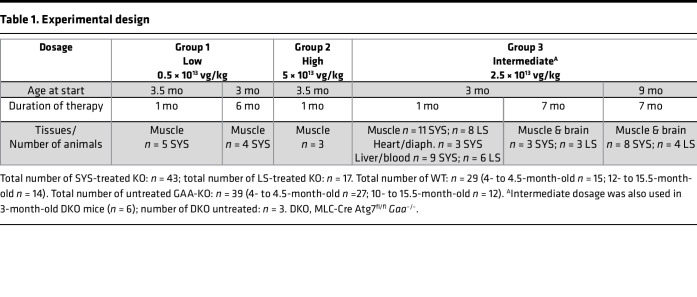
Experimental design

## Version 1 07/18/2023

In-Press Preview

## Version 2 08/22/2023

Electronic publication
