# Supplementary material for: AAV-mediated delivery of secreted acid α-glucosidase with enhanced uptake corrects neuromuscular pathology in Pompe mice
Source: JCI Insight. 2023 Aug 22;8(16):e170199. doi: 10.1172/jci.insight.170199 (PMC10543735; doi:10.1172/jci.insight.170199)
Supplement: Supplemental data [file jciinsight-8-170199-s048.pdf]

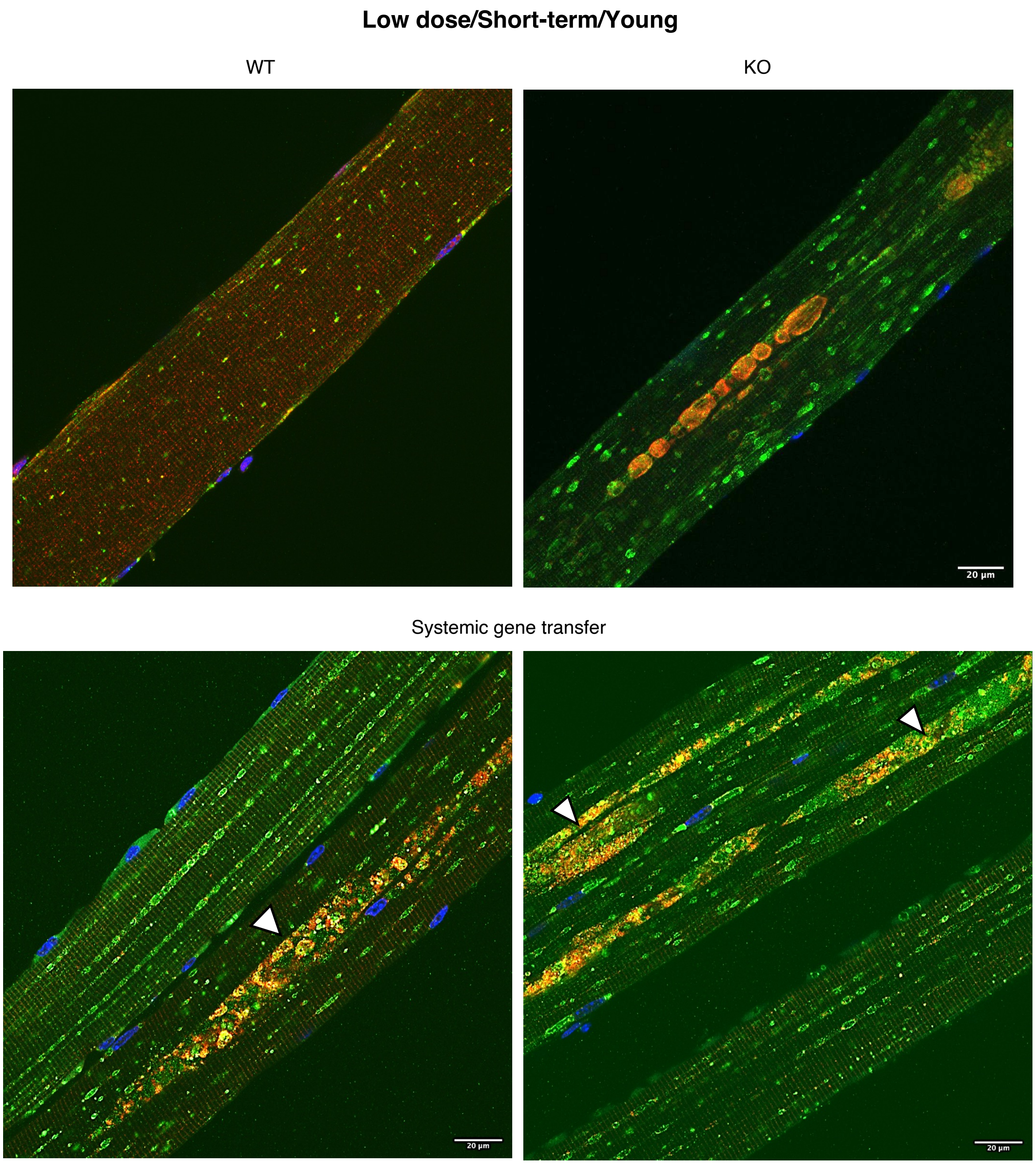

Supplemental Figure 1. **Systemic gene transfer fails to rescue muscle pathology after short-term treatment at a low vector dose.** 3.5-month-old KO mice received a single injection of systemic (SYS) vector at a dose of 0.5×10^{13} vg/kg. The samples were collected one month after dosing. The superficial (pale) part of gastrocnemius muscle was isolated for the experiments. Immunostaining of single fibers with markers for lysosomes (LAMP1; green), autophagosomes (LC3; red), and nuclei (Hoechst dye; blue). Enlarged lysosomes and autophagic buildup (multicolored areas in the core of the fibers) are seen in most fibers from SYS-treated KO mice (Systemic gene transfer; arrowhead); some fibers (~12%) from SYS-treated KO mice are free from autophagic buildup. Bars: 20µm.

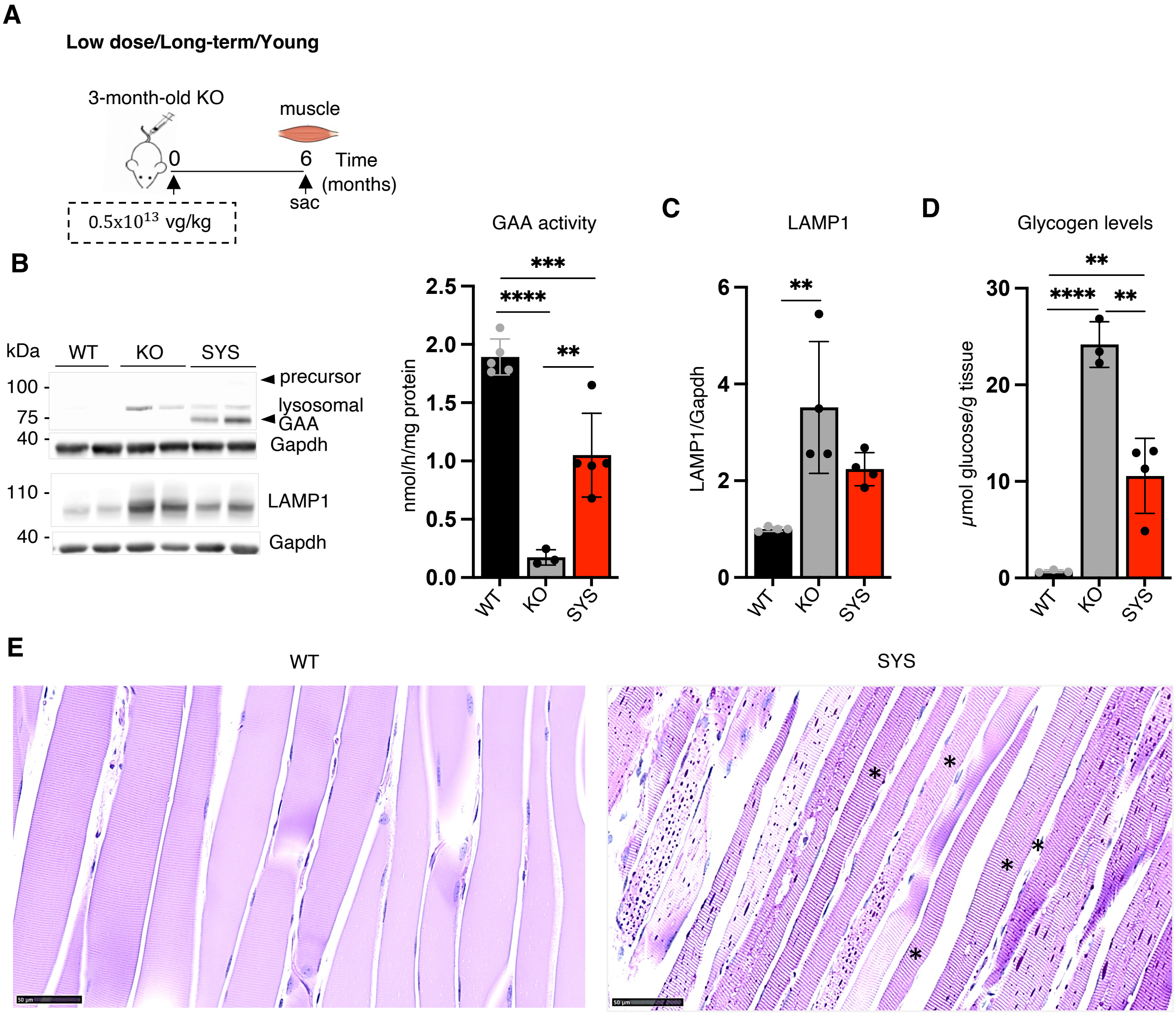

Supplemental Figure 2. **Systemic gene transfer partially rescues muscle pathology after long-term treatment at a low vector dose.** (**A**) Experimental design. 3-month-old KO mice received a single injection of systemic (SYS; n=4) vector at a dose of $0.5x10^{13}$ vg/kg. Age-matched (9-month-old) wild type (WT) and untreated *Gaa*$^{-/-}$ (KO) mice were used as controls. The samples were collected six months after dosing. The superficial (pale) part of gastrocnemius muscle was isolated for the experiments. (**B**) Western blot analyses of whole muscle lysates with anti-human GAA antibody. The mature 76 kDa GAA protein is clearly detectable after long-term treatment. Gapdh was used as a loading control. Graph shows GAA activity in muscle tissues across the groups. GAA activity in muscle from treated KO is below the physiological levels measured in WT mice. (**C** and **D**) The level of lysosomal marker LAMP1 and glycogen content in muscle tissues from SYS-treated KO dropped significantly compared to the values in untreated KO; the effect was more pronounced compared to the short-term (see Figure 1; main text). (**E**) PAS-stained sections of gastrocnemius muscle from WT and SYS-treated mice; multiple fibers from SYS-treated mice appear normal or near normal (asterisks). Bars: 50µm. Statistical significance was determined by one-way ANOVA. Graphs represent mean ± SD. $^{**}p < 0.01$; $^{***}p < 0.001$; $^{****}p < 0.0001$.

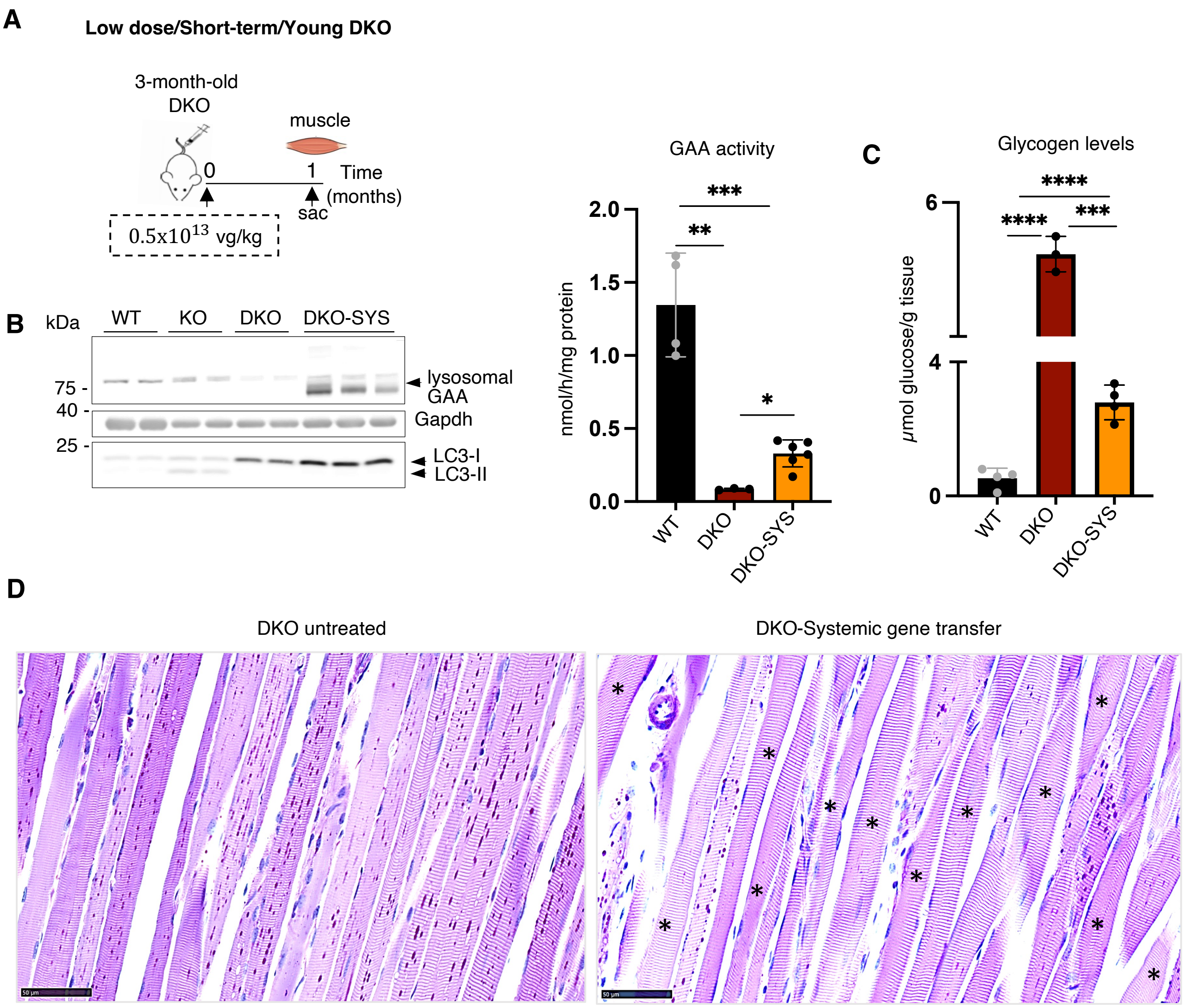

Supplemental Figure 3. Effect of systemic gene transfer in muscle-specific autophagy deficient KO mice (referred to as double knockout; DKO) after short-term treatment at a low vector dose. (**A**) Study design. 3-month-old muscle-specific autophagy-deficient KO mice (referred to as double knockout; DKO) received a single injection of systemic (SYS) vector at a dose of 0.5×10^{13} vg/kg (n=6). Age-matched wild type (WT), untreated KO, and untreated DKO (n=3) mice were used as controls. The samples were collected one month after dosing. The superficial (pale) part of gastrocnemius muscle was isolated for the experiments. (**B**) Western blot analyses of whole muscle lysates with the indicated antibodies. Gapdh was used as a loading control. The LC3-II (an autophagosome membrane–bound LC3 form) was completely absent in muscle lysates from DKO mice, thereby confirming suppression of autophagy. Mature 76 kDa lysosomal GAA is clearly detectable in muscle from SYS-treated DKO (DKO-SYS) mice; GAA activity increased significantly compared to untreated DKO but remained significantly lower compared to the values in the WT. (**C**) Glycogen content in muscle tissues across the groups. (**D**) PAS-stained sections of gastrocnemius muscle from untreated and treated DKO; PAS-positive structures (small dots) unevenly distributed among fibers are seen in a muscle biopsy from an untreated DKO mouse; multiple fibers appear normal or near normal in a muscle biopsy from a treated DKO mouse (asterisks). Bars: 50μm. Statistical significance was determined by one-way ANOVA. Graphs represent mean ± SD. *p < 0.05; **p < 0.01; ***p < 0.001; ****p < 0.0001.

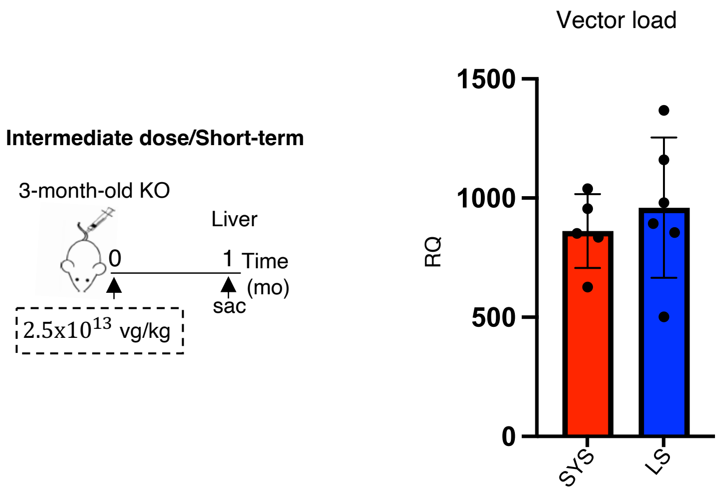

Supplemental Figure 4. Analysis of viral load in the liver. 3-month-old KO mice received a single injection of SYS (n=5) or LS (n=6) vectors at a dose of 2.5x10^{13} vg/kg. The liver samples were collected one month after dosing. Age-matched (4-4.5-month-old) wild type (WT) and untreated *Gaa*$^{-/-}$ (KO) mice were used as controls. The transgene (RBG) was amplified using specific primers and a probe, and the values were normalized by Gapdh gene, which served as an internal control for each sample (relative quantification; RQ). The viral load in the liver was comparable for the two vectors. Statistical significance was determined by one-way ANOVA. Graphs represent mean ± SD.

Intermediate dose/Long-term/Old

Systemic gene transfer

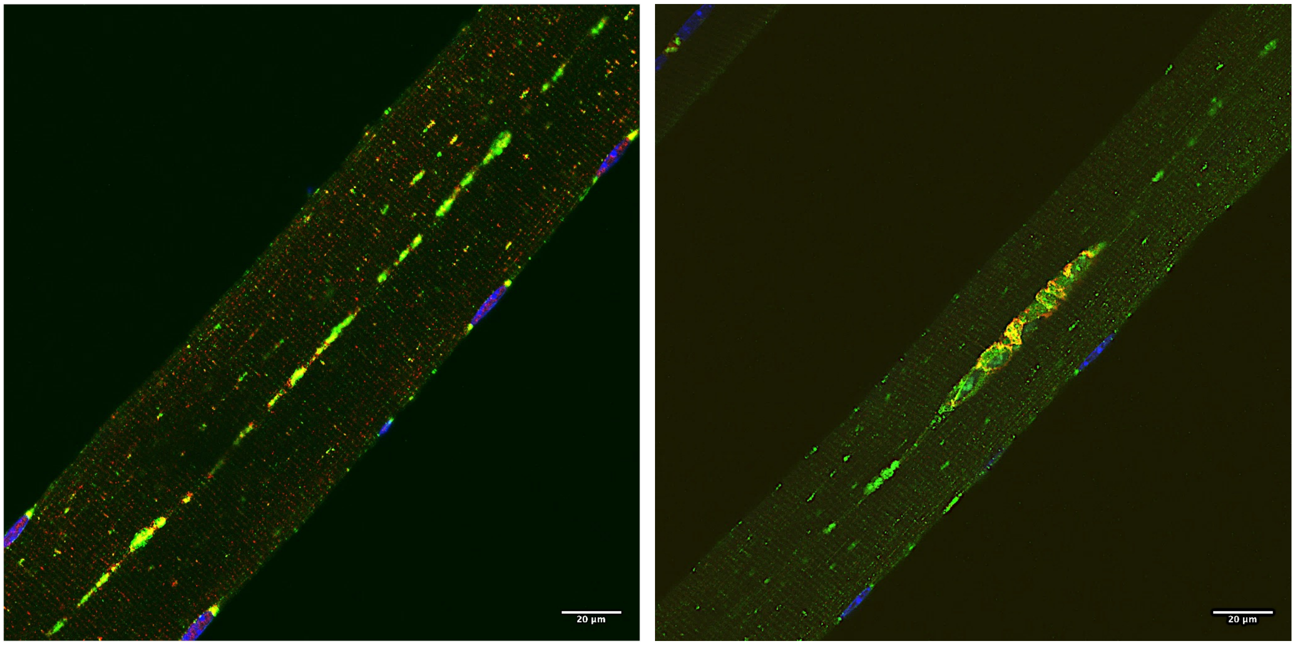

Supplemental Figure 5. Systemic gene transfer reverses muscle pathology in old KO mice. Nine-month-old KO mice received a single injection of systemic (SYS n=8) vector at a dose of $2.5x10^{13}$ vg/kg. Age-matched (15.5-month-old) wild type (WT) and 13.5-month-old untreated *Gaa*$^{-/-}$ (KO) mice were used as controls. The samples were collected 7 months after dosing. The superficial (pale) part of gastrocnemius muscle was isolated for the experiments. Additional images of immunostained single fibers with markers for lysosomes (LAMP1; green), autophagosomes (LC3; red), and nuclei (Hoechst dye; blue); see also Figure 8G in the main text. One of 69 analyzed muscle fibers from SYS-treated KO mice contained a small area of autophagic accumulation (top right panel). Bars: 20µm.

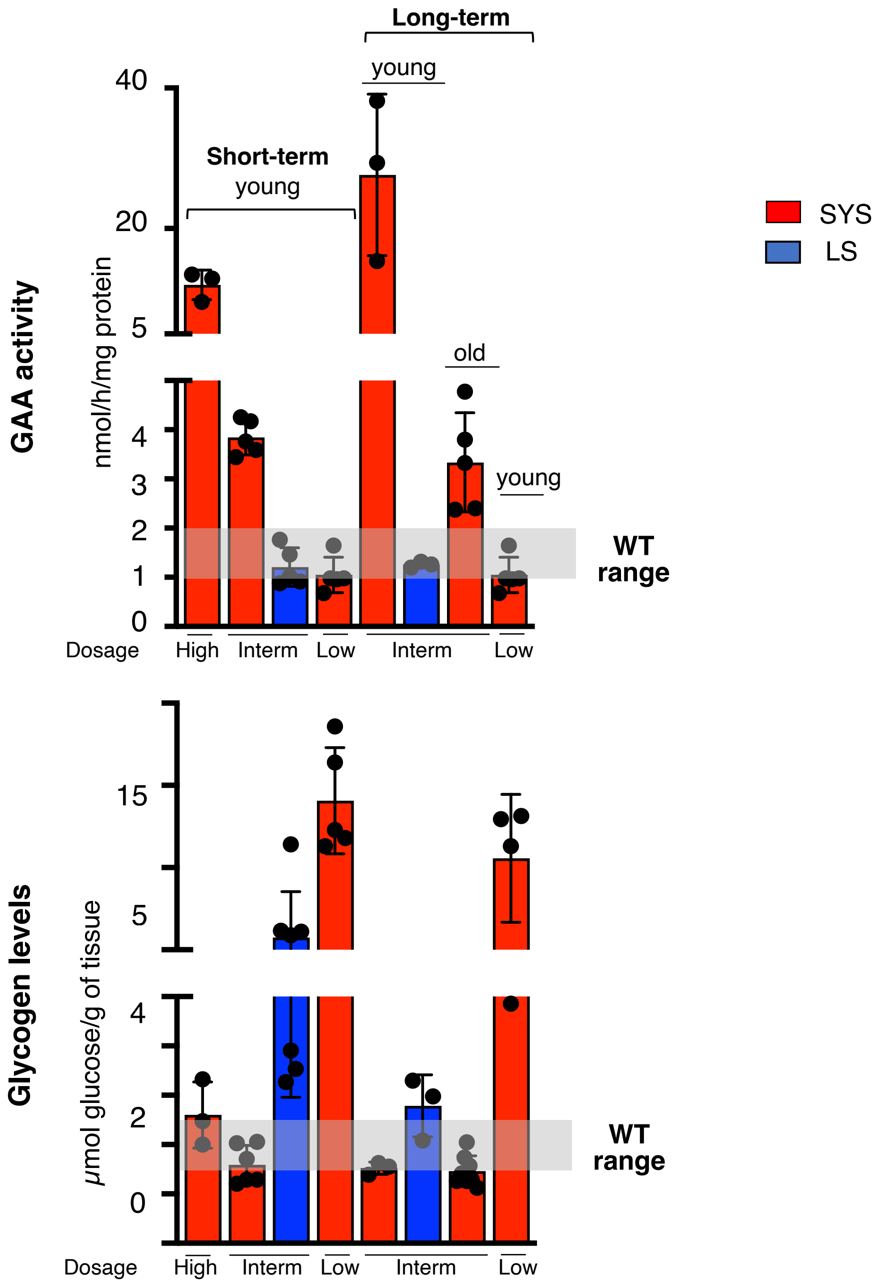

Supplemental Figure 6. Summary of GAA enzyme activity and glycogen content in muscle tissues from all KO mice treated with SYS and LS vectors. Graphs illustrate an inverse relationship between enzyme activity and muscle glycogen content in muscle tissues from treated KO mice. GAA activity in muscle tissues was significantly higher after SYS compared to LS treatment in all experimental conditions. Supraphysiological levels of enzyme activity are required for efficient glycogen reduction to normal levels.

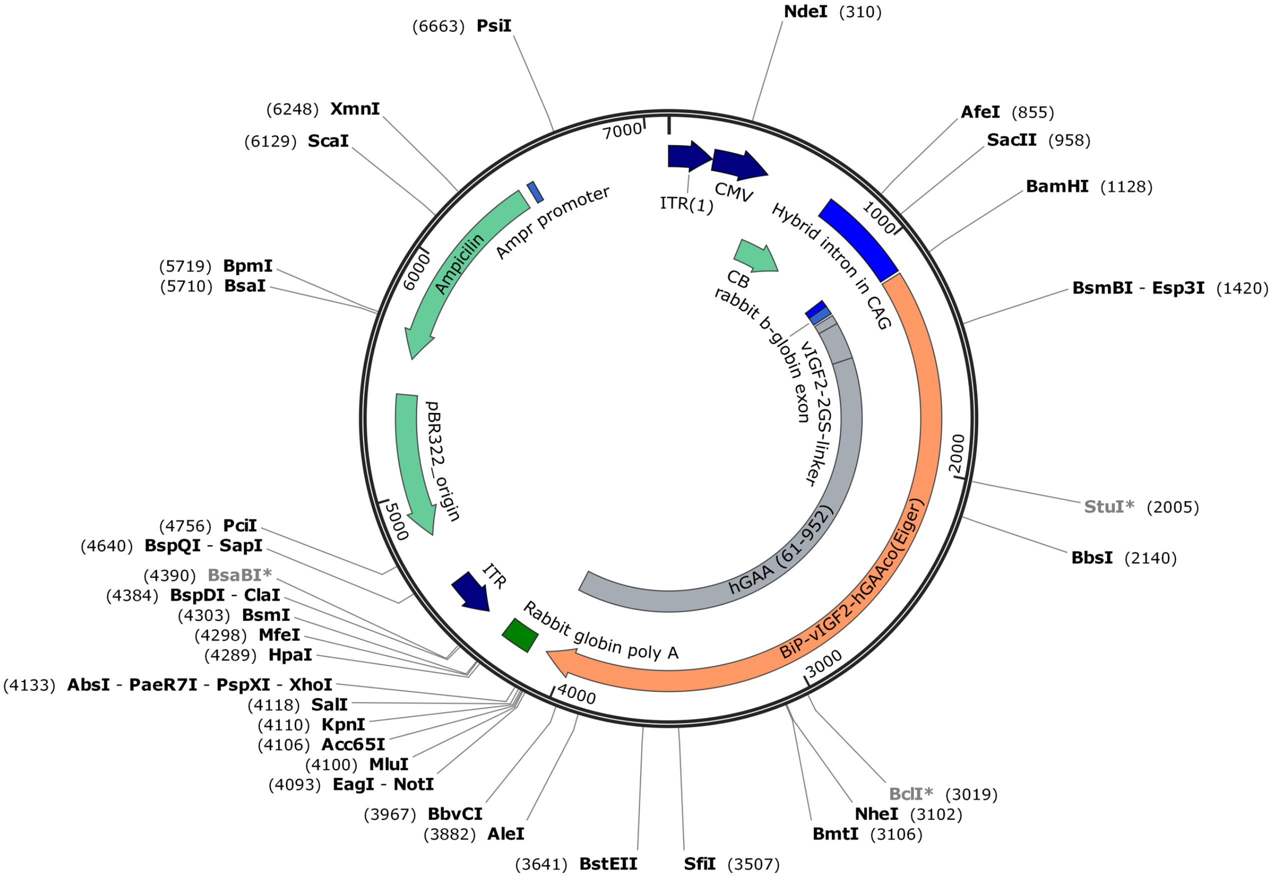

Supplemental Figure 7. Graphical map of the plasmid used to produce systemic (SYS) AAV9.CAG.BiP.vIGF2.hGAAco.rBG.KanR vector. The map was provided by Amicus Therapeutics Inc.

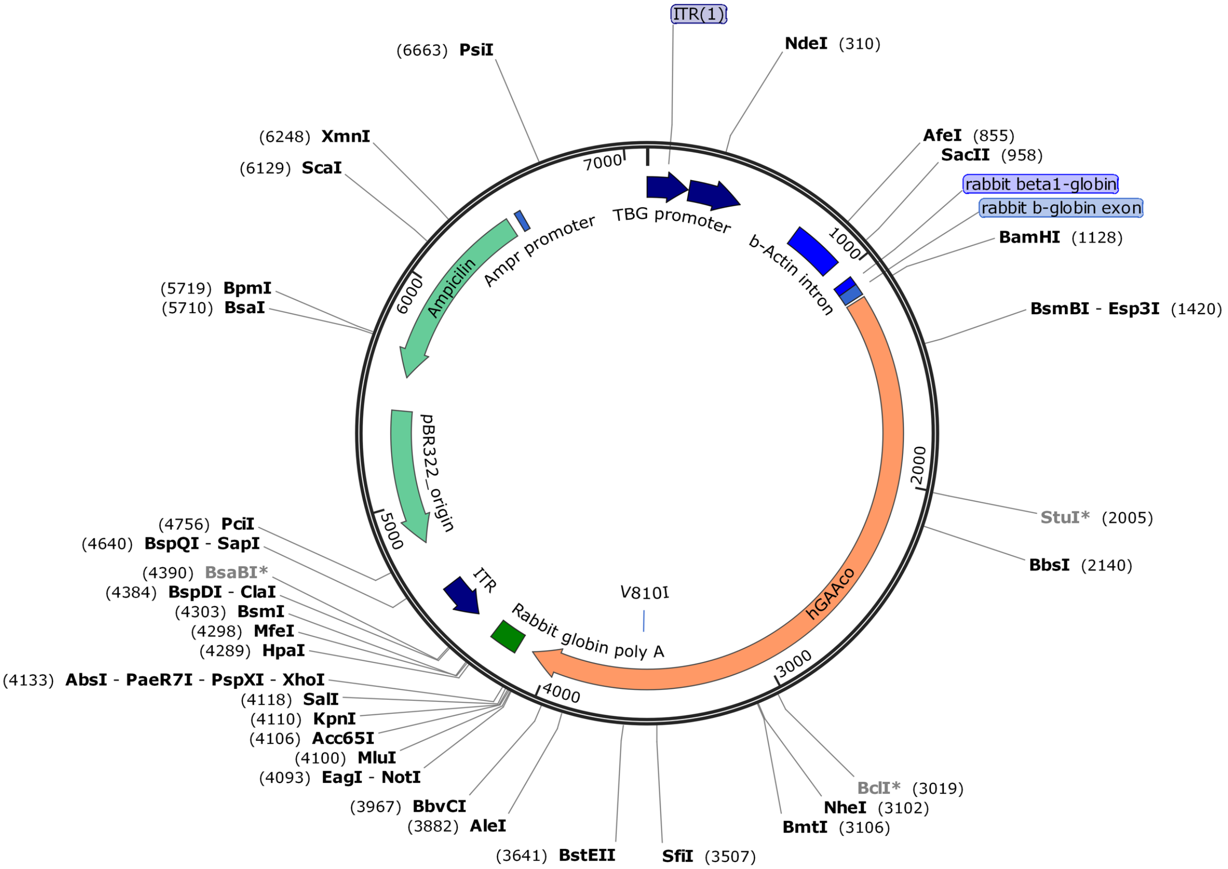

Supplemental Figure 8. Graphical map of the plasmid used to produce liver-secreted (LS) AAV9.TBG.Sp7.delta8hGAAco.rBG vector. The map was provided by Amicus Therapeutics Inc.

Supplemental Table 1. The levels of glucose and liver enzymes following systemic gene transfer

Glucose (mg/dl)				AST (U/L)			
WT	KO	SYS	LS	WT	KO	SYS	LS
184	167	160	144	224	203	70	131
216	116	193	140	178	178	111	161
157	154	190	148	101	157	106	68
195	70	233	169	122	188	158	126
	197	185			435	175	
	232	201			223	326	
		204				105	
**ALT (U/L)**				**AST/ALT**			
WT	KO	SYS	LS	WT	KO	SYS	LS
26	33	18	18	8.62	6.15	3.89	7.28
27	16	20	27	6.59	11.13	5.55	5.96
16	22	23	17	6.31	7.14	4.61	4.00
19	30	26	19	6.42	6.27	6.08	6.63
	36	34			12.08	5.15	
	33	40			6.76	8.15	
		33				3.18	
**Creatine Kinase (U/L)**				**Total Bilirubin (mg/dl)**			
WT	KO	SYS	LS	WT	KO	SYS	LS
748	521	253	522	0.3	0.3	0.3	0.4
596	356	385	935	0.3	0.2	0.4	0.5
249	273	365	199	0.2	0.4	0.5	0.3
428	385	1186	308	0.2	0.5	0.2	0.5
	1178	252			0.3	0.3	
	345	697			0.2	0.4	
		278				0.2	

Supplemental Table 2. Anti-hGAA IgG (ug/ml) in the serum from SYS- and LS-treated KO mice

SYS	LS
0.853	0.607
7.117	0.909
9.235	1.225
1.716	0.899
10.634	0.785
2.453	1.116
10.688	
1.025	
1.773	
